# Dynamic protein coronas revealed as a modulator of silver nanoparticle sulphidation *in vitro*

**DOI:** 10.1038/ncomms11770

**Published:** 2016-06-09

**Authors:** Teodora Miclăuş, Christiane Beer, Jacques Chevallier, Carsten Scavenius, Vladimir E. Bochenkov, Jan J. Enghild, Duncan S. Sutherland

**Affiliations:** 1 Interdisciplinary Nanoscience Center (iNANO), Aarhus University, Gustav Wieds Vej 14, 8000 Aarhus, Denmark; 2 Department of Public Health, Aarhus University, Bartholins Alle 2, 8000 Aarhus, Denmark; 3 Department of Physics, Aarhus University, Ny Munkegade 120, 8000 Aarhus, Denmark; 4 Department of Molecular Biology and Genetics, Aarhus University, Gustav Wieds Vej 10, 8000 Aarhus, Denmark; 5 Department of Chemistry, Lomonosov Moscow State University, Leninskie gory 1/3, 119991 Moscow, Russia

## Abstract

Proteins adsorbing at nanoparticles have been proposed as critical toxicity mediators and are included in ongoing efforts to develop predictive tools for safety assessment. Strongly attached proteins can be isolated, identified and correlated to changes in nanoparticle state, cellular association or toxicity. Weakly attached, rapidly exchanging proteins are also present at nanoparticles, but are difficult to isolate and have hardly been examined. Here we study rapidly exchanging proteins and show for the first time that they have a strong modulatory effect on the biotransformation of silver nanoparticles. Released silver ions, known for their role in particle toxicity, are found to be trapped as silver sulphide nanocrystals within the protein corona at silver nanoparticles in serum-containing cell culture media. The strongly attached corona acts as a site for sulphidation, while the weakly attached proteins reduce nanocrystal formation in a serum-concentration-dependent manner. Sulphidation results in decreased toxicity of Ag NPs.

## Abstract (web summary)

The biomolecule layer adsorbed at the nanoparticle surface and defined as protein corona affects the nanoparticle biophysical properties and functions. Here, the authors suggest that rapidly-exchanging proteins on the outermost layer of the corona modulate sulphidation of silver nanoparticles *in vitro*.

## Graphical abstract

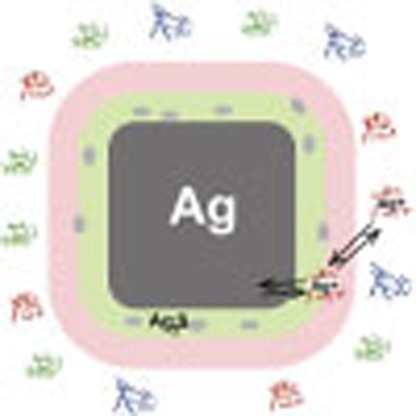

The biological effects of engineered nanomaterials as drug delivery vehicles or as unintentionally released nanoparticles (NPs) are of strong current interest. Biomolecules—mainly proteins—adsorbing at NPs modify their surface properties and are proposed as important modulators of particle–cell interactions^1^,^2^,^3^,^4^,^5^,^6^,^7^,^8^. A pragmatic distinction has been made between the relatively easily studied, strongly attached proteins as long-lived, hard coronas and the weakly attached, rapidly exchanging proteins as soft coronas^9^,^10^,^11^,^12^,^13^. The former are under focus with residence at the particles on timescales relevant for cellular binding and uptake^4^,^6^,^14^, whereas the role of the latter in modulating NP behaviour has yet to be established. Specific and different profiles of molecules concentrated within the hard corona at particles in biological media have been observed for different surface coatings^15^, charges^16^,^17^, sizes^15^,^18^ and shapes^19^. The concept of a biological identity imprinted within the protein corona and which determines NP–cellular interactions^1^,^2^,^3^,^4^,^5^,^6^ has been proposed^20^. Although the long-lived layer has been linked to particle aggregation^19^ and cell association^6^,^14^,^21^, the correlation of protein composition to cellular uptake/toxicity is still relatively weak^4^,^22^,^23^. The involvement of soft corona in physical and/or chemical transformations of particle with potential implications for toxicity is so far unstudied, despite it forming a dense second layer around the strongly attached biomolecules^24^.

In addition to protein corona formation, ion release is central to the toxicity of silver NPs and is an important parameter studied *in vitro*^25^,^26^,^27^ and *in vivo*^28^. Oxidation contributes to ion release through the formation of Ag_2_O on the particles^29^,^30^, which is then dissolved in aqueous media^31^,^32^,^33^. Oxidative dissolution is an important step in Ag_2_S formation from/at Ag NPs^34^. Silver NP sulphidation has been receiving increasing attention, as the resulting sulphide is insoluble in water, decreasing the availability of Ag^+^ and impacting antibacterial^35^ and toxicological^36^,^37^,^38^ effects. After identification of silver sulphide in sewage sludge^39^, interest in studying Ag_2_S was focused on wastewater plants^40^,^41^ and aquatic environments^42^. Although most toxicity experiments are conducted *in vitro*, much less is known about such transformations of Ag NPs under these conditions. Thiols (for example, cysteine) have been proven to bind Ag^+^ in biological environments^43^,^44^. Tracking the oxidation state of intracellular silver showed an evolution from Ag^0^ to oxygen-bound and sulphur-bound Ag ions^45^,^46^. Formation of Ag_2_S in alveolar cells was proposed to explain decreased toxicity of silver nanowires^47^ and their sulphidation in protein-free culture medium was recently studied^48^. A further step involves exploring chemical changes that occur in full culture media, in the presence of protein coronas, before cellular uptake.

Here we demonstrate one clear role for the soft corona in modulating silver NP sulphidation *in vitro*, and highlight the interplay between strongly and weakly attached proteins for the chemical transformation of Ag NPs. We also suggest some potential implications for toxicity, without, however, establishing a clear direct connection between the soft corona and observed toxicological effects. We show for the first time a functional effect of rapidly exchanging proteins, which decreased the amount of nano-Ag_2_S formed at polyvinylpyrrolidone (PVP)-coated Ag NPs incubated in serum-supplemented cell culture media. We propose and study a mechanism for soft corona protein-assisted Ag^+^ transport explaining reduced sulphide formation. Striking differences when going from *in vitro* to *in vivo* relevant protein concentrations are observed and discussed. As it is known that sulphidation decreases silver toxicity^36^,^37^,^38^,^47^,^49^,^50^,^51^, it is not surprising that under conditions where Ag NPs were partially or completely transformed into Ag_2_S in cell culture media, much less toxicity to J774 macrophages and different cytokine secretion profiles are seen compared with silver NPs.

## Results

### Protein coronas modulate nano-Ag_2_S formation at Ag NPs

**Figure 1:**
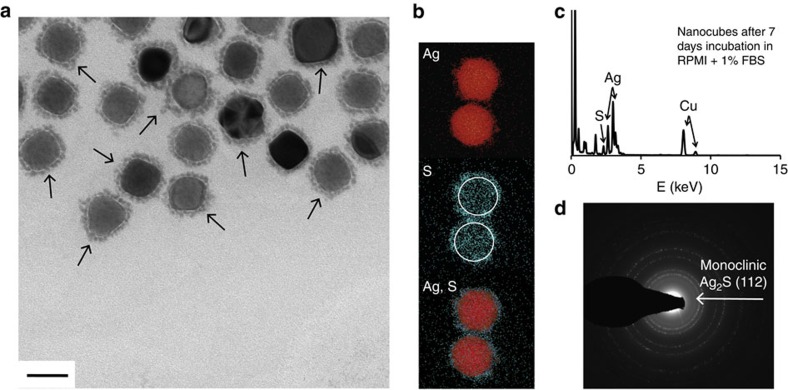
Silver sulphide forms close to the surface of Ag NPs. TEM image with arrows highlighting nano-Ag_2_S (**a**, scale bar 50 nm), X-rays elemental mapping of Ag (red), S (blue, with white rings marking the approximate contour of the Ag NPs) and overlaid Ag and S (**b**), EDS spectrum—with arrows pointing at the peaks corresponding to each element—(**c**) and diffraction pattern—arrow pointing at the diffraction line corresponding to monoclinic Ag_2_S—(**d**) of silver nanocubes after 7 days incubation in RPMI-1640 supplemented with 1% FBS and formation of Ag_2_S at the surface of the Ag NPs.

Upon incubation of PVP-coated, cubic or quasi-spherical Ag NPs in RPMI-1640 cell culture medium supplemented with fetal bovine serum (FBS), new NPs were observed to form close to the surface of the silver. Details regarding incubation are available in the Methods section, Particle incubation in cell culture media subsection. Figure 1a shows a typical transmission electron microscopy (TEM) image of nanocubes after 7 days in 1% serum, with the NPs forming a dispersed layer around the silver core (highlighted by arrows). X-rays elemental mapping (Fig. 1b) and energy-dispersive X-ray spectroscopy (EDS, Fig. 1c) revealed the presence of sulphur. Co-localization of Ag and S matches the small NPs in the proximity of the silver surface (Fig. 1b). The diffraction line at 2.80 (Fig. 1d) corresponds to monoclinic Ag_2_S (ref. 52).

**Figure 2:**
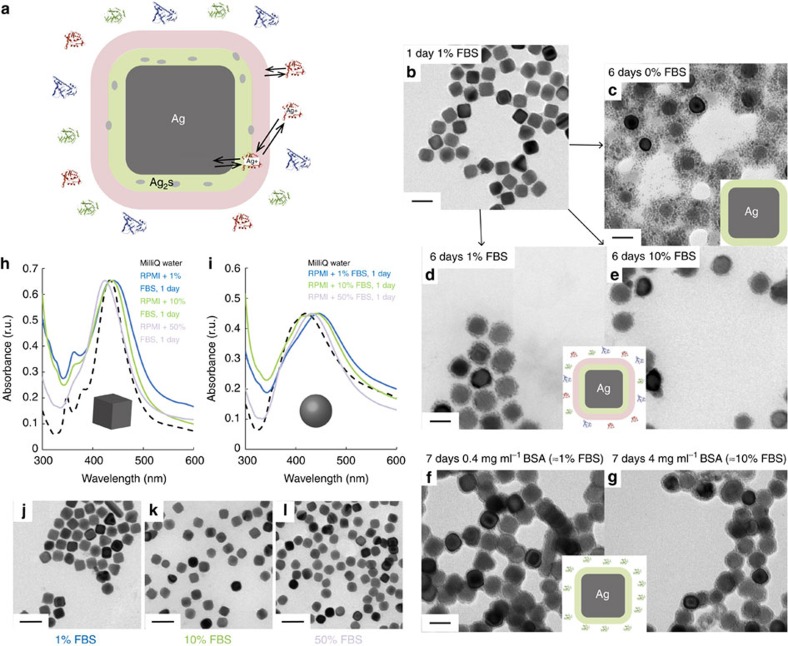
Protein coronas modulate sulphide formation. Proposed mechanism of protein corona-modulated nano-Ag_2_S formation at Ag NPs, with hard corona proteins trapping Ag^+^ released from the nanoparticle surface and soft corona proteins transporting said ions away from the sulphide-formation centres in the long-lived corona (**a**); TEM images of silver nanocubes after 24 h in RPMI-1640 cell culture medium supplemented with 1% FBS (**b**), followed by 6 days incubation in RPMI-1640 with 0% FBS (inset cartoon showing only hard corona around Ag NPs) (**c**), 1% FBS (**d**) or 10% FBS (**e**; common inset cartoon showing hard and soft coronas, as well as free bulk proteins around Ag NPs); TEM images of silver nanocubes after 7 days incubation in RPMI-1640 with 0.4 mg ml^−1^ BSA (**f**) or 4 mg ml^−1^ BSA (**g**) (common inset cartoon showing hard corona and free bulk proteins around Ag NPs); Ultraviolet–visible spectra of cubic (**h**) and quasi-spherical (**i**) Ag NPs after 24 h incubation in RPMI-1640 cell culture medium supplemented with 1, 10 or 50% FBS; TEM images of silver nanocubes after 24 h in RPMI-1640 supplemented with 1% FBS (**j**), 10% FBS (**k**) and 50% FBS (**l**). Scale bars are 100 nm (**b**, **j**–**l**) or 50 nm (**c**–**g**).

When in contact with biological media, NPs get covered with biomolecules^1^,^2^,^3^,^4^. Hard and soft protein coronas around silver nanocubes have previously been quantified and it has been shown that the polymer coating is replaced during the first hour in 1% serum^24^. We observe no sulphide within 1 h, before PVP replacement (Supplementary Fig. 1). The later appearance of Ag_2_S close to the silver surface suggests its formation is related to the layers of adsorbed biomolecules. We hypothesize a mechanism where protein coronas and ion release govern the formation of Ag_2_S at silver NPs (Fig. 2a). We propose that released Ag^+^ can get trapped in the long-lived protein corona where, if enough reduced sulphur and Ag^+^ are available, Ag_2_S may form. In contrast, the rapidly exchanging soft corona proteins prevent sulphide formation by transporting Ag^+^ away from the particle, thus decreasing local ion concentration. To test this hypothesis, one must account for both hard and soft corona proteins, as well as bulk biomolecules.

Silver nanocubes (Supplementary Fig. 2) were incubated in RPMI-1640 with 1% FBS for 24 h (Fig. 2b) to provide a stable hard corona (Supplementary Discussion). The particles were washed, removing unbound and loosely bound proteins while retaining the hard corona (Fig. 2c, cartoon), and re-suspended in RPMI-1640 without serum for 6 days. Similarly, particles were incubated for 7 days in 1 or 10% FBS to ensure the presence of hard and soft coronas, and bulk proteins (Fig. 2d,e cartoon). The same behaviour was seen for quasi-spherical NPs (Supplementary Fig. 3). Ag_2_S is observed under all incubation conditions where serum was present for at least the 24 h needed to fully form the hard corona^24^. Figure 2c–e shows typical TEM images of the different conditions–6 days at 0, 1 and 10% FBS after 24 h in serum-containing media. EDS confirmed the presence of sulphur (Supplementary Fig. 4). Although the total incubation time is the same (7 days) for all samples, the differences in Ag_2_S amounts are striking, with significantly more sulphide observed at the particle surfaces when soft corona and free bulk proteins are absent after the first day. Incubating Ag NPs in serum-containing media for the initial 24 h establishes a long-lived corona, stable after the transition to 0% FBS. It has previously been shown that the hard corona is similar after 24 h in 1 and 10% FBS for different silver nanocubes^24^, and in the present study, we observed the same protein profiles for NPs of various sizes (Supplementary Fig. 5). Here there is a similar long-lived layer on all Ag NPs, as confirmed by mass spectrometry (Supplementary Tables 1–3); what varies (0, 1 and 10% FBS) are the soft corona and the bulk protein concentrations. Nano-Ag_2_S formation at Ag NPs decreased with soft corona presence and the increase in free proteins, with significantly more sulphide at 0% than 1% and 10% FBS. The variation does not appear linear: differences between 1 and 10% FBS are not as striking as between 0 and 1%. Furthermore, although in the presence of serum the particles are dispersed even after 7 days (Supplementary Fig. 6), at 0% FBS the nano-Ag_2_S forms bridges between Ag NPs, which result in particle agglomeration (Fig. 2c).

Both soft corona and bulk proteins could bind Ag^+^; distinguishing between the two categories requires absence of one of them. To test the role of soft coronas, bovine serum albumin (BSA), at concentrations equivalent to 1, 10 or 50% FBS, was used to form a hard corona around silver nanocubes (Supplementary Fig. 7). When only BSA is present in the system, the protein–protein interactions needed to form soft coronas do not occur (Supplementary Fig. 8 and Supplementary Methods); incubation in albumin provides only hard coronas and bulk proteins (Fig. 2f,g, cartoon). Figure 2f,g shows slightly more nano-Ag_2_S formation when BSA concentration is increased from equivalent of 1% FBS to 10% FBS; the opposite phenomenon is observed for serum (Fig. 2d,e). Full sets of TEM images are available (Supplementary Figs 9 and 10); similar results were obtained when using lysozyme instead of BSA (Supplementary Figs 11 and 12). The observed increase in Ag_2_S content at higher albumin/lysozyme concentrations may be due to a thicker, faster-formed hard corona, allowing for trapping and sulphidation of more Ag^+^. Although sulphide appears for incubation in both FBS and BSA/lysozyme, increasing serum concentration—unlike increasing albumin/lysozyme concentration—decreases Ag_2_S formation. This is attributed to the lack of a soft corona when a single type of protein exists in the system^11^—as shown in Supplementary Fig. 8 and Supplementary Methods—as hard coronas and free bulk proteins are present in both cases.

To further study the soft corona influence, experiments were performed at serum concentrations from 1 to 50%, over 24 h. Ultraviolet–visible spectra were collected and plasmon peak shifts were observed. Owing to localized surface plasmon resonances, variations in peak position indicate refractive index changes around particles. Red shifts of 2–4 nm upon binding of proteins to Ag NPs have been seen previously^24^. Here, we observe changes of up to 45 nm indicating the presence of a material with high refractive index—like Ag_2_S—close to the Ag NPs. Comparison to TEM images (Fig. 2j,l and Supplementary Figs 13 and 14) shows the more sulphide present at the surface, the larger the red-shift in the plasmon peaks is. Increased protein concentration and, therefore, soft corona protein content^24^, results in a visible decrease of nano-Ag_2_S formation not just at prolonged incubation, but also after 24 h. Analysis of multiple TEM images revealed almost no sulphide at the NP surface in 50% serum. Furthermore, TEM and ultraviolet–visible data indicate a lower sulphide content after 24 h in 10% compared with 1% FBS. Figure 2h,i shows spectra for cubic and quasi-spherical Ag NPs after 24 h in 1, 10 or 50% serum. Decreasing FBS concentration and, implicitly, the amount of soft corona^24^, results in the formation of more Ag_2_S at Ag NPs, as suggested by the plasmon peak shifted towards higher wavelengths when going from 50% to 10% and 1% serum. NP dissolution (Supplementary Fig. 15) results in reshaping and resizing on a similar timescale for these nanocubes, with concomitant changes of the optical spectra. This leads to the decreased intensity of the signal around 350 nm, where a more prominent peak is characteristic of larger silver nanocubes, with sharper edges^53^. Reduction in particle diameter, coupled with shape changes from cubes to sphere-like, also explains the blue-shift around 435 nm (Fig. 2h) after incubation in 50% serum. No reshaping is observed for the quasi-spherical NPs, while slight diameter decrease may occur.

It has been shown that shifts in plasmon peak position, coupled with finite-difference time-domain simulations of plasmonic behaviour may be used to quantify refractive index changes caused by protein adsorption at Ag NPs^24^. Via a similar approach (Supplementary Figs 16 and 17, Supplementary Table 4 and Supplementary Methods) we estimate that, at the Ag NP concentration used here (10 μg ml^−1^), 15–40% of the silver is transformed into sulphide.

### Ion release from Ag NPs is necessary for nano-Ag_2_S formation

**Figure 3:**
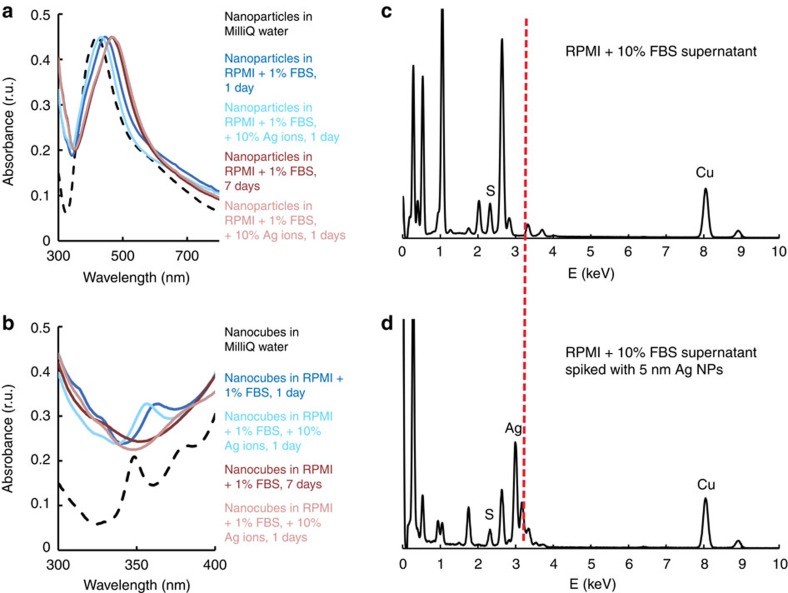
Silver nanoparticle dissolution is involved in nano-Ag_2_S formation. Ultraviolet–visible full spectra of quasi-spherical Ag NPs (**a**) and quadrupole peak detail of nanocubes (**b**) incubated (1 day: blue or 7 days: pink) in RPMI-1640 cell culture medium supplemented with 1% FBS, with or without added extra 10% (by mass) Ag^+^ ions from AgNO_3_; EDS spectra of the supernatant obtained after centrifugation of Ag NPs incubated for 7 days in RPMI-1640 with 1% FBS, before (**c**) and after (**d**) spiking with 5 nm PVP-coated Ag NPs, with dotted red line highlighting the presence of a silver signal only in the spiked sample.

Sulphidation of silver NPs involves the presence of ionic silver therefore variations in Ag^+^ content may influence the formation of Ag_2_S. To test this parameter, AgNO_3_—with Ag^+^ representing 10% by weight of the particulate silver—was added to the NP suspension at the beginning of 1 or 7 days incubation in RPMI-1640 with 1% FBS (Supplementary Figs 18–21 for 10% serum). ultraviolet–visible spectra of quasi-spherical particles with or without extra ions were collected (Fig. 3a); a less pronounced red-shift was seen when free Ag^+^ were added, especially at short incubation, suggesting a slower NP dissolution in the presence of extra ions.

Nanocubes have several characteristic plasmon peaks, of which a quadrupole (≈350 nm) and a dipole (≈435 nm; Supplementary Fig. 18). The quadrupole indicates the degree to which a NP is cubic, as well as its size^53^, and, as such, it is used to track the synthesis of silver nanocubes^54^ (Supplementary Fig. 22). Dissolution reduces particle size and blunts cube edges, resulting in flattening of the quadrupole peak (Fig. 3b). After 7 days in 1% FBS, the signal disappears almost completely.. At 24 h, both samples exhibit a pronounced quadrupole peak, with a visibly sharper signal when 10% free Ag^+^ were added at the beginning of the incubation. The results suggest Ag NPs in samples with added ions undergo slower and, perhaps, less dissolution, observable in resizing and reshaping of the particles. This behaviour could be explained by the existence of a plateau ion concentration in the bulk, as previously observed^32^,^45^. Adding free ions may decreases the amount of Ag^+^ required from NP dissolution for achieving this equilibrium concentration, but further investigations would be necessary to elucidate the mechanism.

Inspection of multiple TEM images suggests there is no difference in the amount of Ag_2_S at silver NPs with or without added 10% Ag^+^ at 7 days, but some decrease in sulphide incidence occurs at 24 h (Supplementary Fig. 19). This indicates the formation of nano-Ag_2_S requires the release of Ag^+^ from NPs and not just the existence of free ions in the bulk. The observation is strengthened by control experiments with silica particles in RPMI-1640 with FBS and free silver ions; no nano-Ag_2_S was detected after 7 days (Supplementary Fig. 23).

Atomic absorption spectroscopy (Supplementary Fig. 15) confirmed the presence of silver in the bulk, strengthening the soft corona ion transport mechanism; it did not, however, indicate the chemical form of the silver. Although the timescale of soft corona exchange is on the order of seconds and minutes and nano-Ag_2_S formation occurs over hours, we verified that at prolonged incubation the rapidly exchanging proteins do not transport Ag_2_S nano-crystals away from the Ag NP surface. We measured EDS spectra and mapped supernatants collected after 7 days incubation in 1% FBS, before and after spiking in PVP-coated Ag NPs (Fig. 3c,d and Supplementary Fig. 24) of similar size (5 nm) and amount to that of nano-Ag_2_S. If Ag_2_S nanocrystals were transported to the bulk, a peak would appear in the EDS spectrum around 3 keV. Absence of this signal in the un-spiked supernatant indicates nano-Ag_2_S is not transported by the soft corona.

### Sulphur sources and Ag/S ratio influence nano-Ag_2_S formation

**Figure 4:**
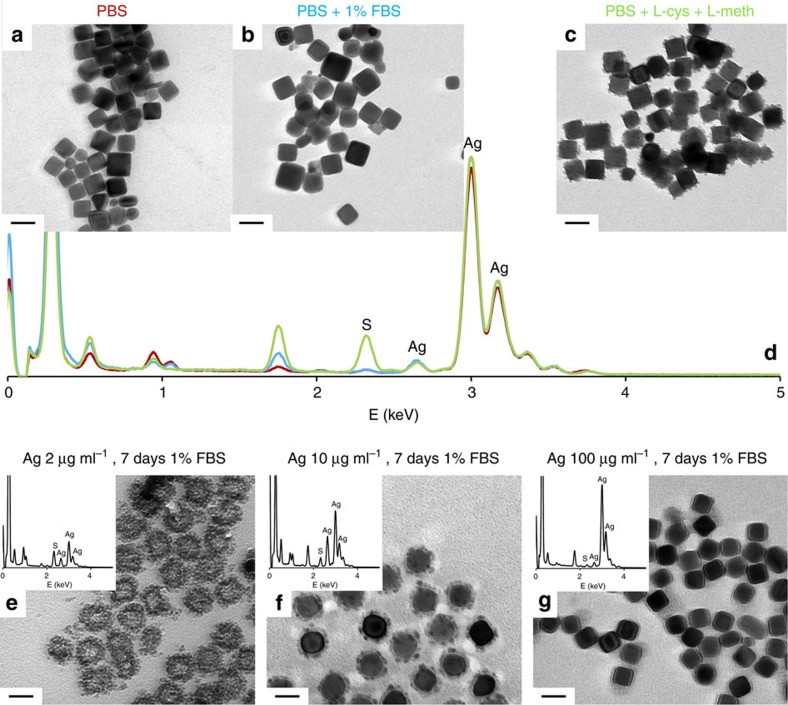
Sulphur sources and the Ag:S ratio influence Ag_2_S formation. TEM images of Ag NPs after 7 days incubation in PBS (**a**), PBS supplemented with 1% FBS (**b**) and PBS supplemented with L-cysteine and L-methionine at the same concentrations of amino acids as those found in RPMI-1640 (**c**) and corresponding EDS spectra (**d**); TEM images and corresponding EDS spectra (insets) of Ag NPs after 7 days incubation in RPMI-1640 supplemented with 1% FBS, with initial silver concentrations of 2 μg ml^−1^ (**e**), 10 μg ml^−1^ (f) and 100 μg ml^−1^ (**g**), with elemental mapping images provided in Supplementary Fig. 26. Scale bars are 100 nm (**a**–**c**) or 50 nm (**e**–**g**).

Sulphur-containing gases can contribute to the formation of Ag_2_S at Ag NPs exposed to air, but this is a lengthy and slow process, extended over 24 weeks^55^. In the liquid phase, RPMI-1640 provides several sulphur sources (Supplementary Tables 5 and 6), with L-cysteine and L-methionine accounting for most of the reduced S; furthermore, many serum proteins contain cysteine residues. To pin-point the sulphur responsible for Ag_2_S formation in our case, we incubated Ag NPs in phosphate-buffered saline (PBS) supplemented with either 1% FBS or L-cysteine and L-methionine at the same concentrations as in RPMI-1640. After 7 days, no sulphide was seen in PBS or in buffer with serum (Fig. 4a,b), but nano-Ag_2_S was present in the amino-acid-supplemented buffer (Fig. 4c and Supplementary Fig. 25). EDS spectra (Fig. 4d) confirmed the observations from TEM images. These experiments show reduced sulphur from small molecules and not from proteins is the one forming Ag_2_S.

We further tested the effect of Ag/S ratios by varying the amount of nanocube stock suspension added to a given volume of serum-supplemented RPMI-1640. After 7 days, increasing the initial silver concentration from 2 to 10 and then to 100 μg ml^−1^ (Fig. 4e–g and Supplementary Fig. 26) drastically decreases Ag_2_S formation by limiting the L-cysteine and L-methionine available per Ag^+^. *In vivo* cysteine concentration is more than double that in RPMI, making more S available for Ag_2_S formation^56^,^57^. At the lowest Ag/S ratio, the particles are almost entirely transformed into Ag_2_S, forming ‘pockets' of sulphide that conserve the shape of the initial particle, with the metal core still visible in some cases (Fig. 4e). These observations suggest sulphidation is confined to the protein hard corona, in agreement with EDS showing the absence of nano-Ag_2_S from the suspension supernatant (Fig. 3c,d).

### Protein corona-mediated sulphidation impacts cell toxicity

**Figure 5:**
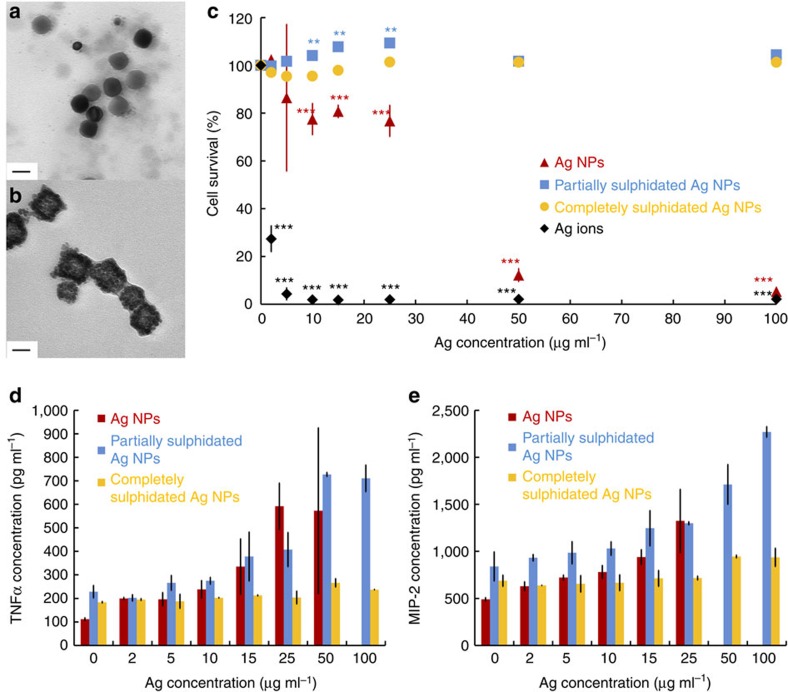
Corona-mediated sulphidation of Ag NPs impacts particle toxicity. TEM images of partially sulphidated Ag NPs after pre-incubation in RPMI-1640 with 10% FBS (**a**) and completely sulphidated Ag NPs after pre-incubation in RPMI-1640 with 1% FBS (**b**); scale bars are 50 nm; Viability of J774 murine macrophages (as measured with MTT assays) after 24 h exposure to various concentrations (2, 5, 10, 15, 25, 50 and 100 μg ml^−1^) of Ag^+^ ions (black diamonds), pristine Ag NPs (red triangles), partially sulphidated Ag NPs (blue squares) and completely sulphidated Ag NPs (orange circles); error bars are provided as standard deviation; statistically significant differences (two-tailed *t*-test, with all data sets showing normal distribution and similar variance values) as compared with the control are marked with **P<0.005 or ***P<0.0005 (*n*=6), with all the *P* values available in Supplementary Table 7 (**c**); release profiles of TNFα (**d**) and MIP-2 (**e**) after 24 h exposure of J774 macrophages to various concentrations (2, 5, 10, 15, 25, 50 and 100 μg ml^−1^) of pristine (red), partially sulphidated (blue) and completely sulphidated (orange) Ag NPs; the missing concentrations of TNFα and MIP-2 after exposure to pristine Ag NPs are above the measuring limit (see calibration curves in Supplementary Fig. 29).

Previous research has already shown that trapping Ag^+^ in the form of insoluble Ag_2_S decreases the toxicity of silver^50^,^51^ and Ag NPs^35^,^49^, mostly in the case of aquatic environments^36^,^37^,^58^ and in soil^38^, which are settings with low protein contents, but higher concentrations of other components that are not prevalent under *in vitro* cell study conditions. Although the effects under *in vitro* parameters have not been studied to the same extent, some information is also available on the decreased toxicity of sulphidated Ag NPs to cultured cells^47^. Our results confirm such already published findings and extend the observations to a cell line where the consequences of sulphidation have not previously been investigated. We indirectly tested the effects of corona-mediated sulphidation on the toxicity of Ag NPs to J774 macrophages by exposing cells to partially and completely sulphidated NPs (obtained by pre-incubation in 10 and 1% FBS respectively, Fig. 5a,b) to mimic *in vitro* conditions, as well as NPs with no sulphide, to mimic *in vivo* settings (Supplementary Fig. 27). Detailed experimental procedures are available in the Methods section and in Supplementary Methods. Ag NPs disrupted mitochondrial activity, as measured using a 3-(4,5-dimethylthiazol-2-yl)-2,5-diphenyltetrazolium bromide (MTT) assay, and caused cell death at concentrations above 25 μg ml^−1^, whereas partially and completely transformed particles showed no effect at concentrations as high as 100 μg ml^−1^ (Fig. 5c). Silver ions were lethal to the cells even at the lowest concentration (2 μg ml^−1^), but pristine, partially and completely sulphidated Ag NPs were suitable for analysis of potential effects at sub-lethal doses. Of all the molecules measured in the cell supernatants, responses above the detection limit were seen for interleukin-1beta, interleukin-6, interleukin-18, tumour necrosis factor alpha (TNFα) and macrophage inflammatory protein 2 (MIP-2; Supplementary Fig. 28 and Fig. 5). Granulocyte–macrophage colony-stimulating factor was also measured at the highest particle doses in the Ag NPs samples, but for most systems, granulocyte–macrophage colony-stimulating factor values were below the detection limit and, therefore, they are not included here. The most pronounced impact is observed on TNFα and MIP-2. TNFα is a cytokine released by macrophages at early inflammatory stages together with interleukin 6 (ref. 59), which we also observed (Supplementary Fig. 28). A threefold increase in its production is caused by pristine and partially sulphidated Ag NPs, but not by completely transformed ones (Fig. 5d). This result indicates upregulation of TNFα production by Ag requires the presence of nano-particulate or ionic silver, as previously seen^60^,^61^. A similar trend (Fig. 5e) was observed for MIP-2, a cytokine involved in cell recruitment to the site of infection following the initiation of the inflammatory response^62^. Together, these results confirm that sulphidation, which we show is mediated by the dynamic protein coronas and is more likely to occur at the serum concentrations used *in vitro* than *in vivo*, decreases the toxicity of silver NPs both at lethal and sub-lethal doses. However, although we show, for the first time, a link between both soft and hard protein coronas and Ag NPs sulphidation and confirm previous findings connecting sulphidation to decreased toxicity, we cannot, at this time, provide a direct link between the soft corona and the diminished toxicological effects of sulphidated Ag NPs. Furthermore, technical limitations in conducting *in vitro* studies at different FBS concentrations do not allow us to account for the Ag^+^ transported by soft corona proteins in the bulk of the pre-incubation system. As silver ions are known for their toxicity and our experimental setting does not permit investigation of their interaction with cells when bound to rapidly exchanging proteins, we cannot make a general claim about the overall toxicological impact that the soft corona-mediated biotransformation of Ag NPs has under various *in vitro* or *in vivo* scenarios.

## Discussion

We have shown for the first time a situation where the weakly attached protein layer forming the soft corona has a visible and measurable effect on the transformation of Ag NPs in a complex biological environment. We demonstrated the presence of crystalline nano-Ag_2_S at the surface of silver NPs upon incubation in cell culture medium. Reduced sulphur, an organic layer at the Ag NPs and release of ions from the metal core are necessary for sulphide formation. Protein concentration greatly impacted the amount of nano-Ag_2_S observed at the particles through a soft corona protein-assisted mechanism of Ag^+^ removal. In the absence of a rapidly exchanging corona, the decrease in sulphide formation upon increased bulk protein concentration was no longer observed, in agreement with the proposed mechanism. Greater free silver ion concentrations introduced in the media did not increase sulphide formation. We have studied well-defined Ag nanocubes and quasi-spherical particles formed via PVP stabilization. Although we expect the proposed mechanism to apply to Ag NPs having other sizes, shapes and coatings, the particulars of the experimental situation—such as incubation time, media, Ag_2_S formation rate and crystal size, protein concentration—are likely to influence the specific outcomes.

The low water solubility of Ag_2_S decreases Ag^+^ ions bioavailability and, although this phenomenon has been studied extensively in ecotoxicology settings^32^,^37^,^39^,^40^,^41^,^42^, little is known about the effect of NP sulphidation in cell culture media^48^. Ions are an important component in silver toxicity to cells; transformations impacting their availability should be taken into account when analysing the stability of Ag NPs in protein-containing media relevant for *in vitro* experiments and interpreting subsequent toxicity studies. We show that even partial sulphidation of Ag NPs prevents cell death, whereas complete sulphidation also prevents the increased pro-inflammatory cytokines production seen with pristine particles. The observed decrease in Ag_2_S formation at increased protein contents may raise a question regarding using *in vitro* results to predict *in vivo* scenarios, as the bulk biomolecule concentration in the latter settings is much higher than in the former. However, for this to become an issue, a direct link between the dynamic protein coronas and the toxic effects of Ag NPs should first be established in future research. Further studies into the bioavailability and effects of soft corona-bound Ag^+^ from particle dissolution in protein-containing media are also necessary to obtain a clear and full picture of how biomolecules-modulated biotransformations may change NPs' toxicity effects *in vitro* and *in vivo*.

## Methods

### Particle synthesis and characterization

Silver NPs, both cubic and quasi-spherical, were prepared using the polyol method^63^, where particle shape is controlled by the ratio between the capping agent (PVP) and the silver precursor^64^. Briefly, a specific amount of silver trifluoroacetate dissolved in anhydrous ethylene glycol is reduced by ethylene glycol at high temperature (145–155 °C) in the presence of PVP (Mw≈55,000 Da). The ratio of PVP to CF_3_COOAg dictates the outcome regarding particle shape. For the cube synthesis, trace amounts of HCl and NaSH·xH_2_O are added to the reaction mixture, as described elsewhere^63^. The particles were purified by repeated washing with acetone, ethanol and MilliQ water^24^. The synthesis was tracked by collecting ultraviolet–visible spectra (Shimadzu UV-visible-NIR spectrophotometer, UV-3600) of the reaction mixture (a few drops in MilliQ water) at various times (Supplementary Fig. 22). The resulting silver particles were quantified using flame atomic absorption spectroscopy (F-AAS, PerkinElmer Analyst 300 atomic absorption spectrometer mounted with a silver lumina hollow cathode lamp), after digestion in 65% HNO_3_. NP size was obtained using the SPIP scanning probe image software (Image Metrology) to analyse TEM images of at least 500 particles.

### Particle incubation in cell culture media

RPMI-1640 (Invitrogen) is a medium widely used for cell cultures, including for the J774 murine macrophages employed here. We incubated Ag NPs (cubic or quasi-spherical) in RPMI-1640 with or without added supplements of heat-inactivated FBS (HyClone; 1–50% by volume), for 1 or 7 days. As we are studying a model system from the perspective of a mechanism of chemical transformation, our choice of serum concentrations and incubation times is only partially based on real toxicology settings. As such, 10% FBS is used as the typical serum supplement for *in vitro* toxicity studies^65^,^66^,^67^, with 24 h being the go-to time for acute toxicity experiments^26^,^45^,^65^,^66^,^67^,^68^,^69^. However, *in vitro* Ag NPs exposure studies have been performed at lower serum concentrations^45^,^68^,^69^, down to 1% (ref. 25), which is the concentration selected in our work. Furthermore, the 1% provides a better model system to study and understand mechanisms, as the low protein concentration makes the protein exchange-dependant process at the NP surface take longer, with fewer biomolecules available to participate. Although FBS contents higher than 10% are not common for *in vitro* studies, they are closer to an *in vivo* situation, so 50% serum was selected to better investigate the behaviour of Ag NPs in a realistic setting. However, we perform a mechanistic study on model systems, therefore the incubation times were chosen to have a fully formed hard corona on Ag NPs^24^ (24 h) and to observe an extensive chemical transformation of the NPs (7 days), even though the onset of the sulphidation occurs, as we show, much earlier and clear changes are visible at 24 h. To study the influence of free ions on nano-Ag_2_S formation, AgNO_3_ (Sigma-Aldrich) aqueous solutions (10% Ag^+^ by weight of the total Ag NP mass) were added to the media at the beginning of the incubation process for some of the samples.

### Sample analysis post-incubation

Ultraviolet–visible spectra were collected against a MilliQ water background, over a wavelength domain of 300–800 nm. The peak positions of the resulting spectra were assessed using the Savitzky–Golay method to derive the spectral plots in the SpecManager software (ACD Labs). After incubation, free proteins were eliminated by centrifugation of particles in a Heraeus Multifuge X1R table top centrifuge (Thermo Scientific) and removal of the supernatant. The silver ions in the supernatants were quantified through F-AAS. Ag NPs were washed with MilliQ water three times through re-dispersion of the pellets and TEM/STEM samples were prepared by dropping 5–10 μl of suspension on a formvar/carbon-supported copper grid (Ted Pella) and leaving it to dry overnight. Imaging and diffraction studies were performed using a Phillips CM20 transmission electron microscope working at 200 keV. EDS and X-ray elemental mapping experiments were performed on a Talos 200X STEM instrument.

### EDS and elemental mapping of supernatants

Silver nanocubes were incubated (10 μg ml^−1^) for 7 days in RPMI-1640 cell culture medium supplemented with 1% FBS. The resulting sample was centrifuged to pellet the particles. A drop of the supernatant was deposited on a formvar/carbon-supported TEM copper grid and left to dry overnight. 150 μl of the remaining supernatant was separated from the pelleted particles, moved to a clean Eppendorf tube and spiked with 40 μl of PVP-coated NanoXact 5 nm Ag NPs (nanoComposixm, stock concentration 20 μg ml^−1^). The NanoXact Ag NPs used for spiking are comparable in size to the observed Ag_2_S nanocrystals and the amount added provides a mass of silver comparable to that in the Ag_2_S nanocrystals formed at the Ag NPs. A drop of the spiked supernatant was deposited on a TEM grid and left to dry overnight. EDS spectra and elemental maps from the resulting samples were collected using a Talos 200X STEM machine.

### Cell line

The J774A.1 murine macrophage cell line (referred to as J774) was obtained from Cell Line Service (#400220). The cells were cultured in RPMI-1640 cell culture medium supplemented with penicillin (100 μg ml^−1^), streptomycin (100 U ml^−1^), GlutaMAX (1 ×) and 10% heat-inactivated FBS. The cells were kept in at 37 °C humidified atmosphere with 5% CO_2_. The cell culture medium and all the supplements were purchased from Gibco.

### Particle pre-incubation and toxicity studies

Pristine Ag nanocubes, as well as partially and completely sulphidated Ag nanocubes were used for toxicity testing. Silver NPs (2 μg ml^−1^) were pre-incubated in RPMI-1640 supplemented with 10 or 1% FBS, resulting in either their partial or their complete transformation into Ag_2_S (Supplementary Fig. 27). After pre-incubation, the samples were centrifuged, the supernatants were removed and the pelleted particles were re-suspended in RPMI-1640 with 10% FBS. Re-suspension was done in such a way as to up-concentrate the samples to the desired concentrations, namely 2, 5, 10, 15, 25, 50 and 100 μg ml^−1^ silver. For each of the final concentrations, pre-incubation was done in separate tubes. For the partially and completely transformed Ag NPs, up-concentration was done correcting for the amount of silver lost through ion release during pre-incubation. The resulting samples were fed to the J774 cells. Dosing of the cells was performed in a blinded experimental setting with the researcher performing the toxicity studies not being informed of the specific content of each tube provided for cell treatment. Cytokine production was quantified using a multiplex assay and cell mitochondrial activity was measured using an MTT assay (Supplementary Methods) in six separate replicates. Sample sizes down to three are routinely used in MTT assays. We believe larger sample numbers (six in this case) only serve to strengthen the significance of the results. Values are calculated as mean±standard deviation.

### Data availability

The authors declare that the data supporting the findings of this study are available within the article and its supplementary information files.

## Additional information

**How to cite this article:** Miclăuş, T. *et al*. Dynamic protein coronas revealed as a modulator of silver nanoparticle sulphidation *in vitro*. *Nat. Commun.* 7:11770 doi: 10.1038/ncomms11770 (2016).

## Supplementary Material

Supplementary Information Supplementary Figures 1-29, Supplementary Tables 1-7, Supplementary Discussion, Supplementary Methods and Supplementary References
