# Supplementary material for: Dynamic protein coronas revealed as a modulator of silver nanoparticle sulphidation in vitro
Source: Nat Commun. 2016 Jun 9;7:11770. doi: 10.1038/ncomms11770 (PMC4906166; doi:10.1038/ncomms11770)
Supplement: Supplementary Information — Supplementary Figures 1-29, Supplementary Tables 1-7, Supplementary Discussion, Supplementary Methods and Supplementary References [file ncomms11770-s1.pdf]

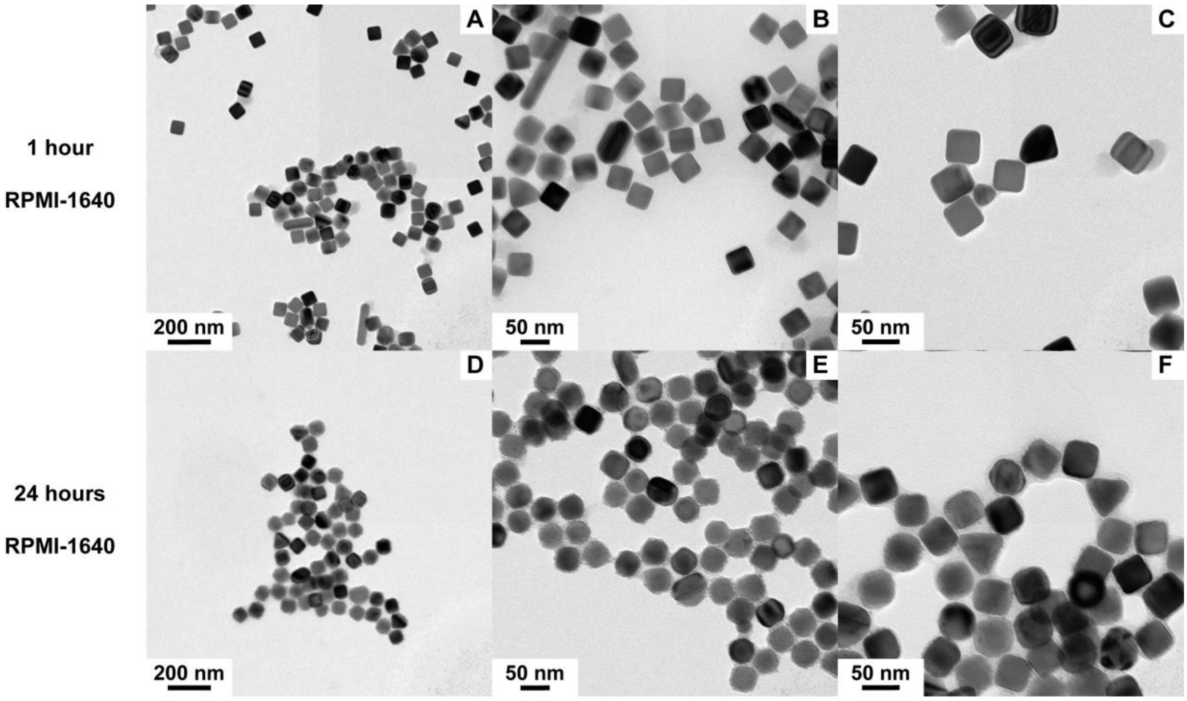

Supplementary Figure 1. Silver nanocubes in serum-free media. TEM images of silver nanocubes incubated in RPMI-1640 without serum for 1 hour **(A-C)**, or 24 hours **(D-F)**. Scale bars are 200 nm **(A, D)** or 50 nm **(B, C, E, F)**

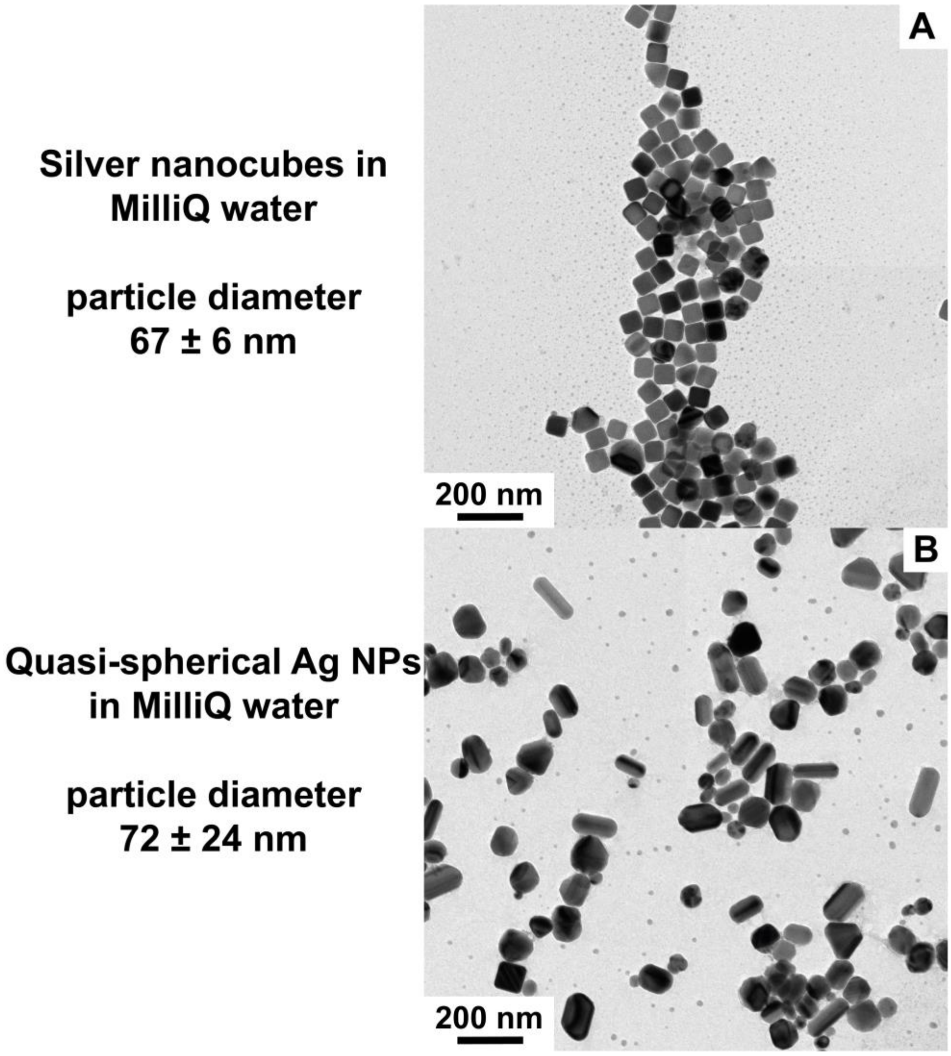

Supplementary Figure 2. Stock silver nanoparticles. Typical TEM images of stock silver nanocubes **(A)** and quasi-spherical silver NPs **(B)** in MilliQ water, with particle diameters calculated using the SPIP^{TM} Image Analysis software (Image Metrology) and analysing at least 500 individual particles from different areas of the TEM grid. Scale bars are 200 nm

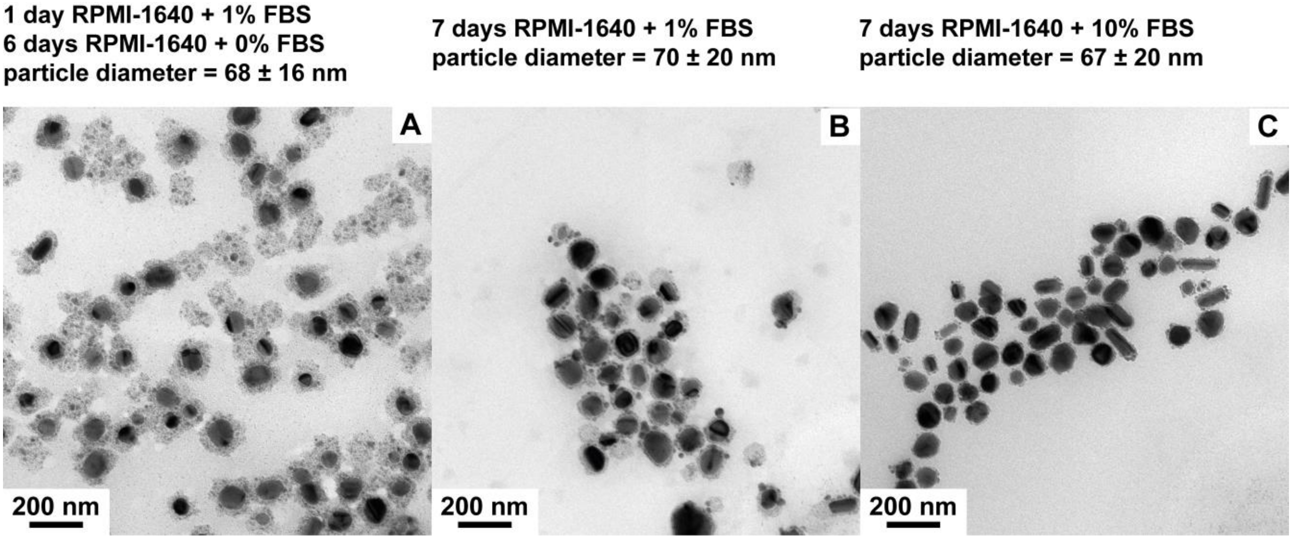

Supplementary Figure 3. Quasi-spherical silver NPs in cell culture media. TEM images of quasi-spherical silver NPs after incubation in RPMI-1640 cell culture medium supplemented with 1 % FBS for 1 day followed by RPMI-1640 without serum for 6 days **(A)**, RPMI-1640 supplemented with 1 % FBS for 7 days **(B)**, and RPMI-1640 supplemented with 10 % FBS for 7 days **(C)**, with the corresponding particle diameters above the images calculated using SPIP^{TM} scanning probe image software from at least 500 individual particles. Scale bars are 200 nm

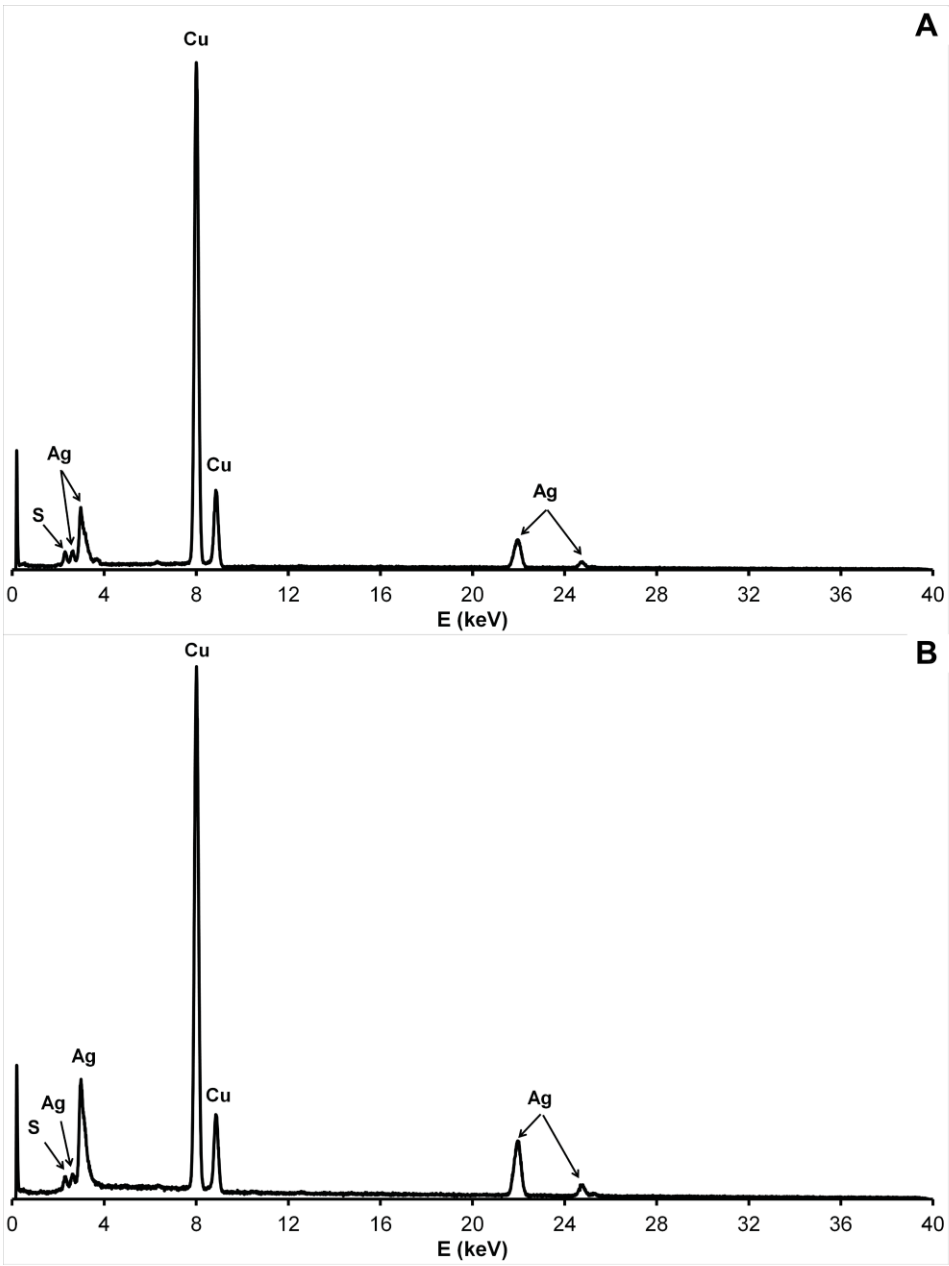

Supplementary Figure 4. EDS spectra of silver NPs in cell culture media. Typical EDS spectra after 7 days incubation of silver nanocubes **(A)** or quasi-spherical Ag NPs **(B)** in RPMI-1640 cell culture medium supplemented with serum for at least the first 24 hours of incubation.

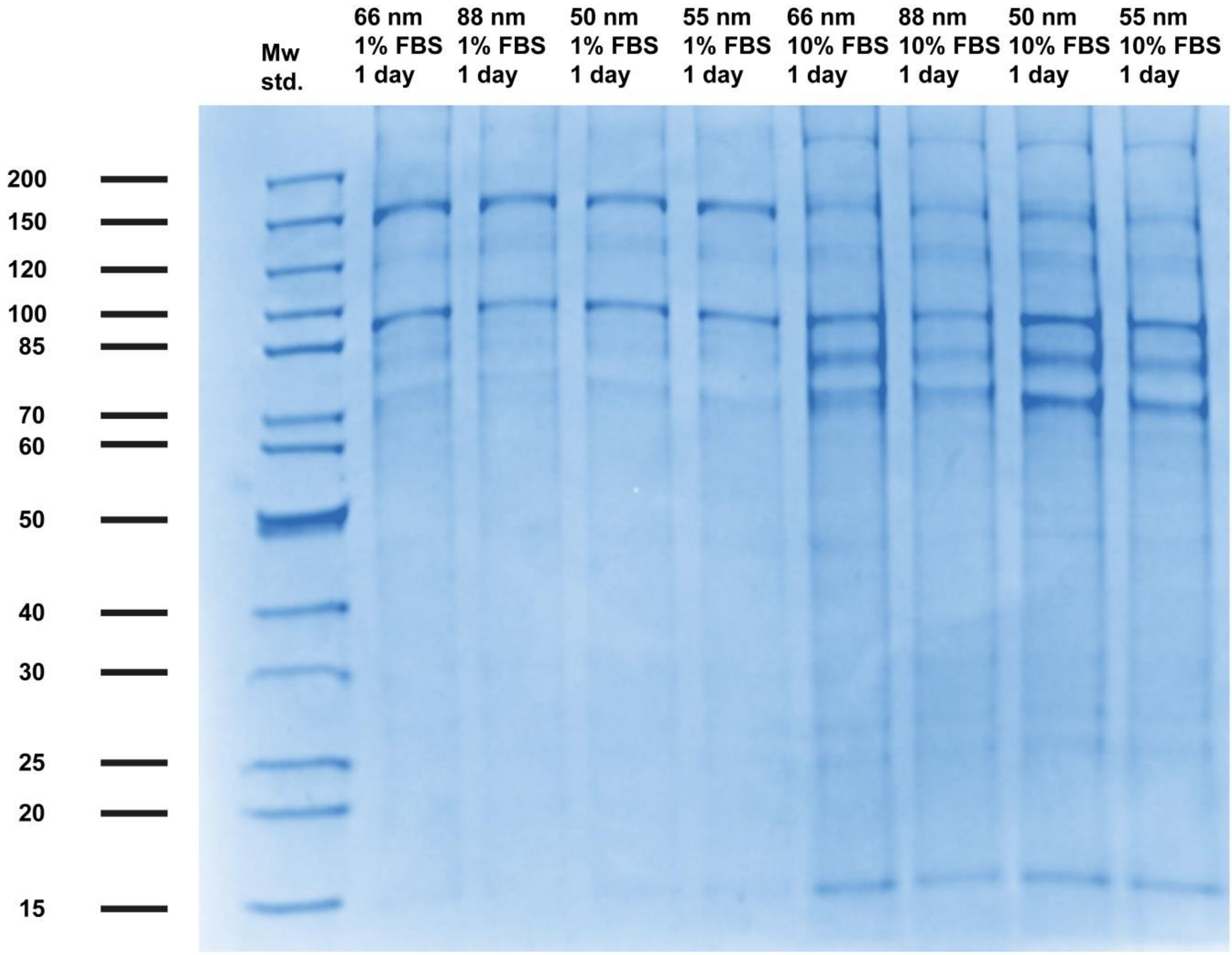

Supplementary Figure 5. Protein hard coronas around silver nanocubes. SDS-PAGE analysis of the hard coronas from silver nanocubes of various sizes incubated for 24 hours in RPMI-1640 supplemented with either 1 % or 10 % FBS. Cube diameter and incubation conditions are listed above each lane. The first lane of the gel pertains to the molecular weight standard.

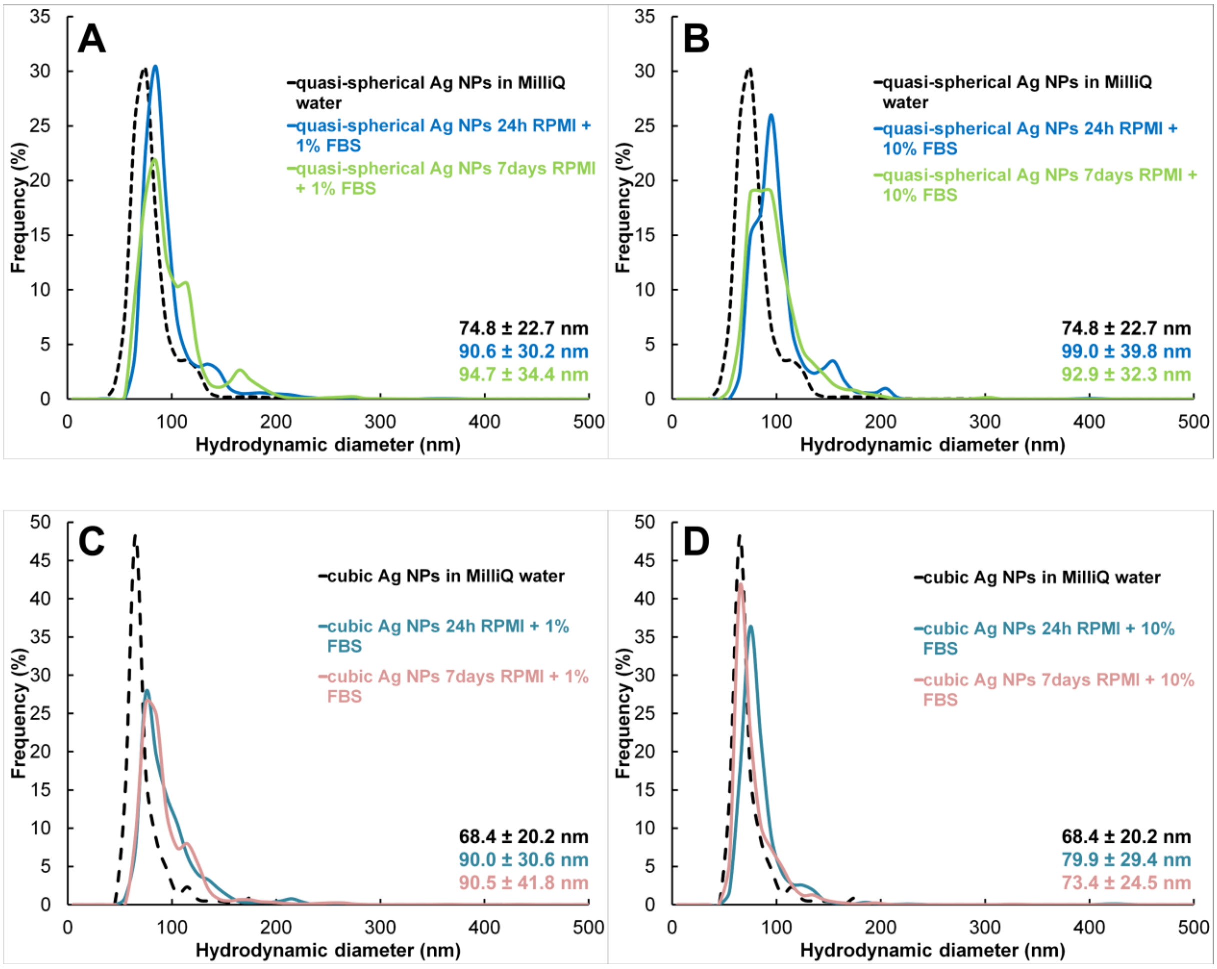

Supplementary Figure 6. Particle stability with Nanoparticle Tracking Analysis. Hydrodynamic diameter of quasi-spherical **(A, B)** and cubic **(C, D)** Ag NPs after 24 hours and 7 days incubation in RPMI-1640 supplemented with 1 % or 10 % FBS. The values on each graph correspond to average diameter ± standard deviation (n=3).

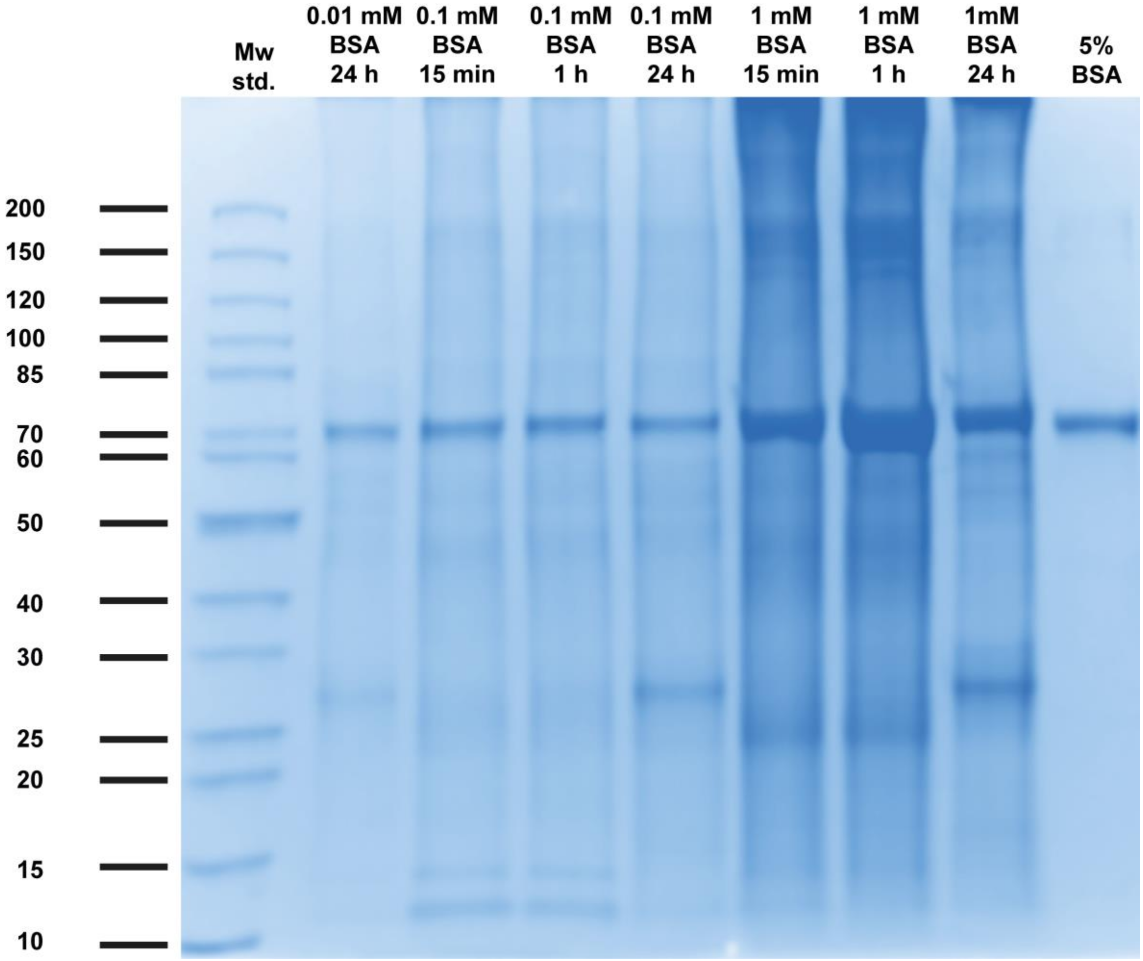

Supplementary Figure 7. BSA hard coronas. SDS-PAGE analysis of the bovine serum albumin hard coronas formed around silver nanocubes at various BSA concentrations and incubation times.

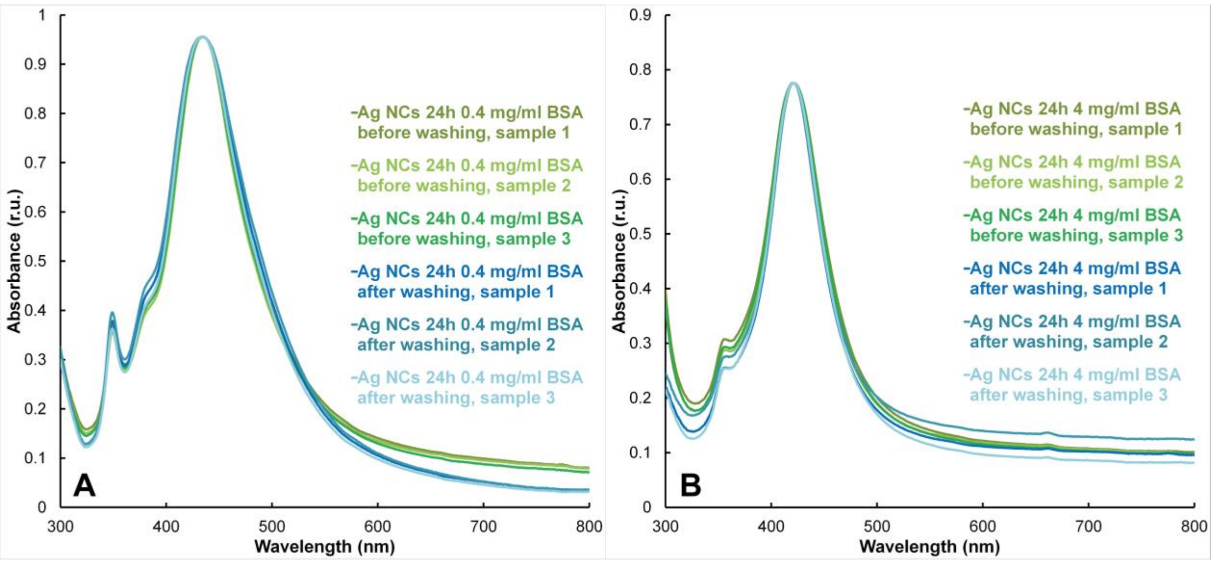

Supplementary Figure 8. Plasmonic response of silver nanocubes in BSA. UV-vis spectra of silver nanocubes before (green) and after (blue) washing following 24 hours incubation in 0.4 mg ml^{-1} BSA **(A)** or 4 mg ml^{-1} BSA **(B)**.

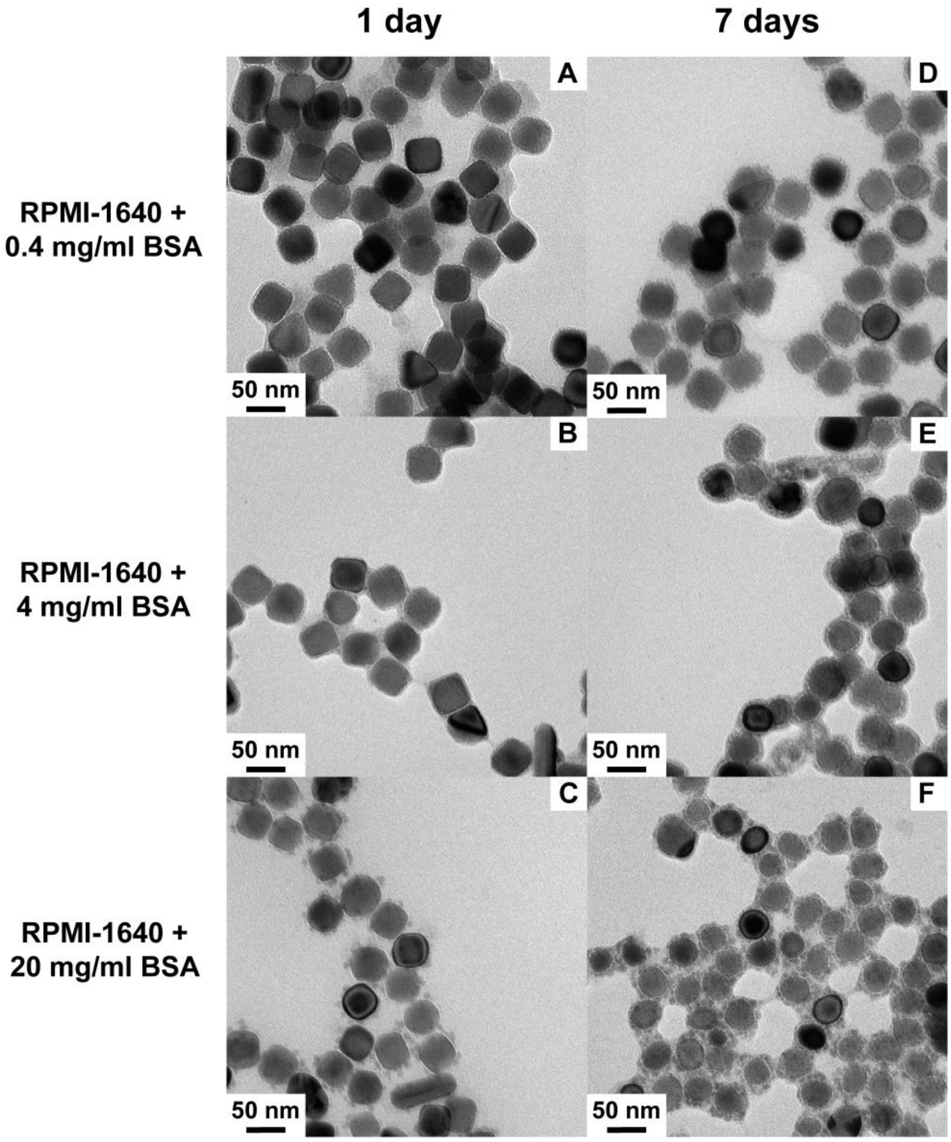

Supplementary Figure 9. Silver nanocubes after incubation in BSA. TEM images of silver nanocubes after 1 day incubation in RPMI-1640 supplemented with 0.4 mg ml^{-1} BSA **(A)**, 4 mg ml^{-1} BSA **(B)** and 20 mg ml^{-1} BSA **(C)** and after 7 days incubation in RPMI-1640 supplemented with 0.4 mg ml^{-1} BSA **(D)**, 4 mg ml^{-1} BSA **(E)** and 20 mg ml^{-1} BSA **(F)**. Scale bars are 50 nm

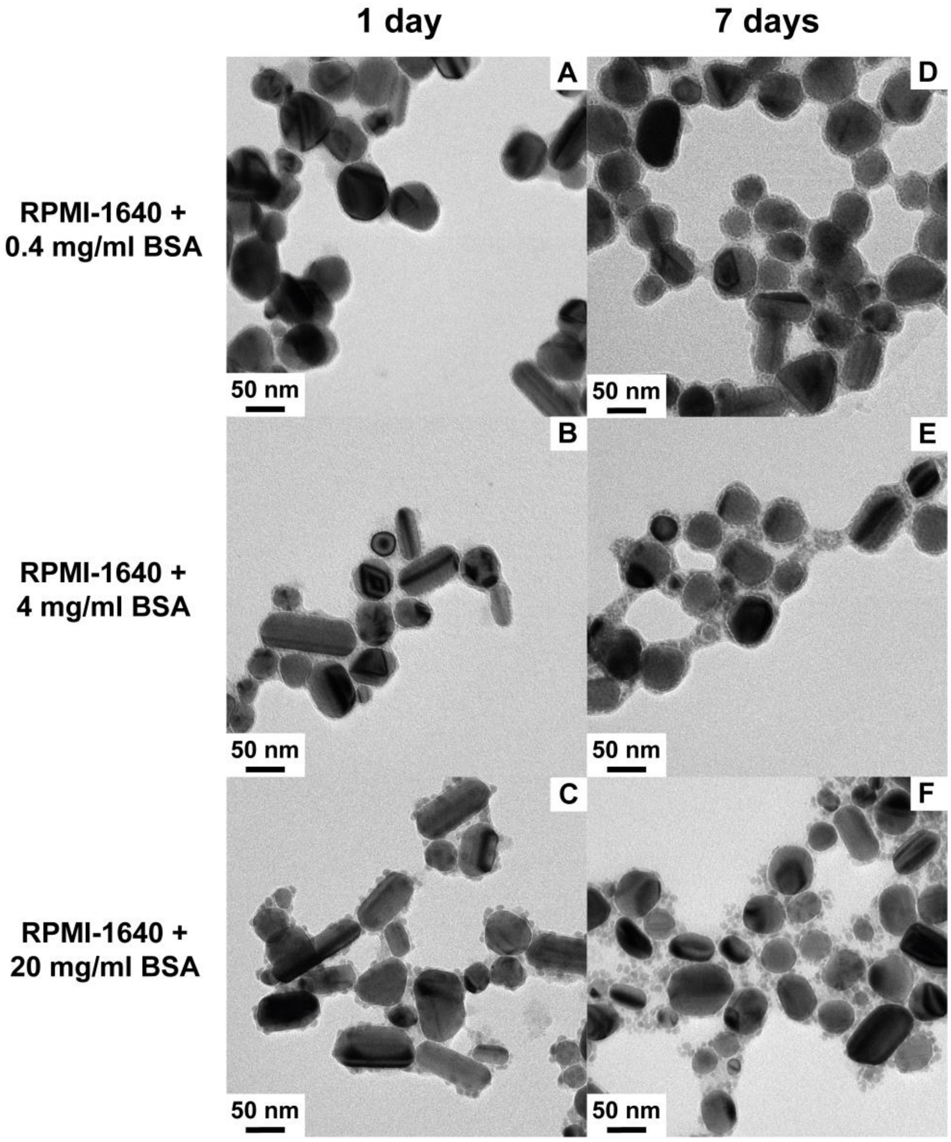

Supplementary Figure 10. Quasi-spherical silver NPs after incubation in BSA. TEM images of quasi-spherical Ag NPs after 1 day incubation in RPMI-1640 supplemented with 0.4 mg ml^{-1} BSA **(A)**, 4 mg ml^{-1} BSA **(B)** and 20 mg ml^{-1} BSA **(C)** and after 7 days incubation in RPMI-1640 supplemented with 0.4 mg ml^{-1} BSA **(D)**, 4 mg ml^{-1} BSA **(E)** and 20 mg ml^{-1} BSA **(F)**. Scale bars are 50 nm

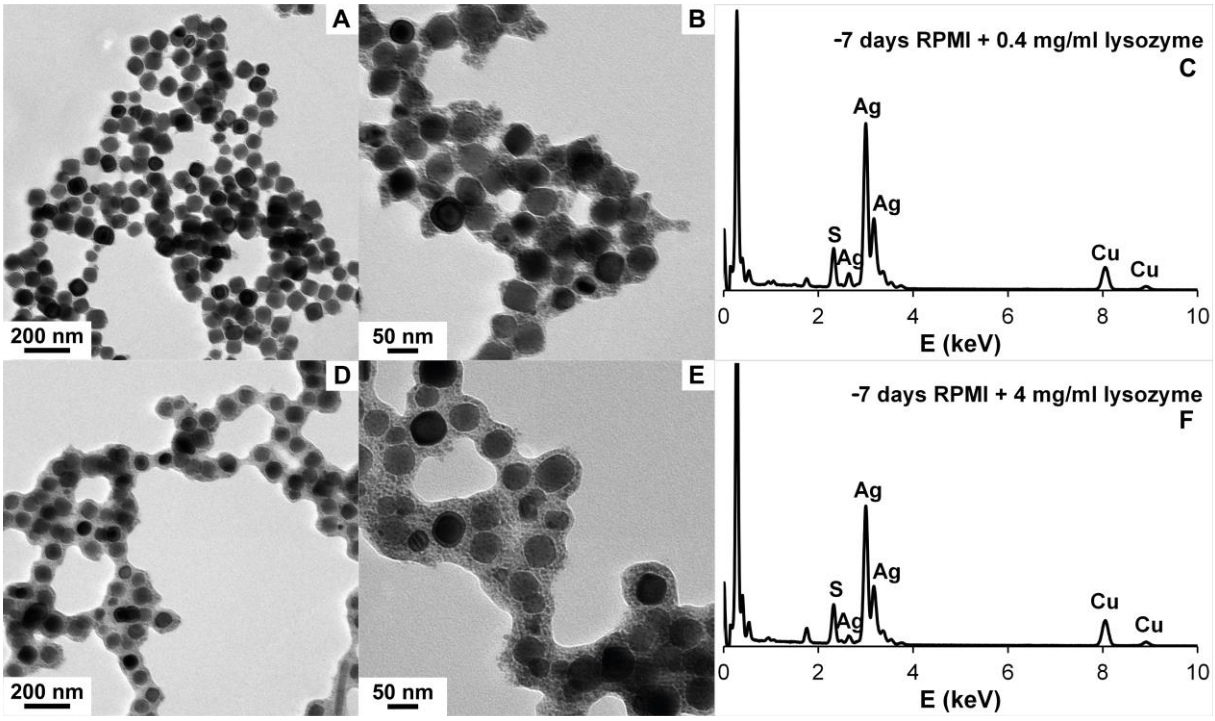

Supplementary Figure 11. Silver nanocubes after incubation in lysozyme. TEM images of silver nanocubes after 7 days incubation in RPMI-1640 supplemented with 0.4 mg ml^{-1} lysozyme **(A, B)** and 4 mg ml^{-1} lysozyme **(D, E)** and corresponding EDS spectra **(C, F)**. Scale bars are 200 nm **(A, D)** or 50 nm **(B, E)**.

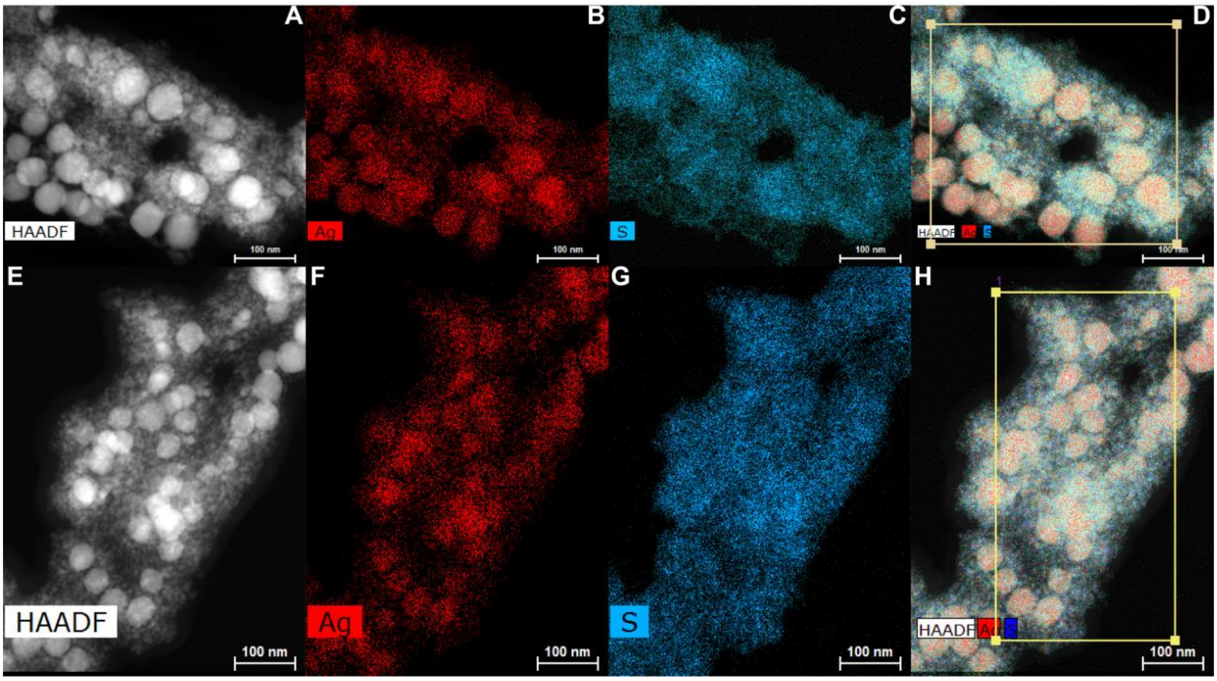

Supplementary Figure 12. Silver NPs after incubation in lysozyme. TEM high-angular annular dark-field **(A, E)**, silver **(B, F)**, sulphur **(C, G)** and overlapped **(D, H)** elemental mapping of Ag NPs after 7 days incubation in RPMI-1640 supplemented with 0.4 mg ml^{-1} lysozyme and 4 mg ml^{-1} lysozyme respectively. Scale bars are 100 nm.

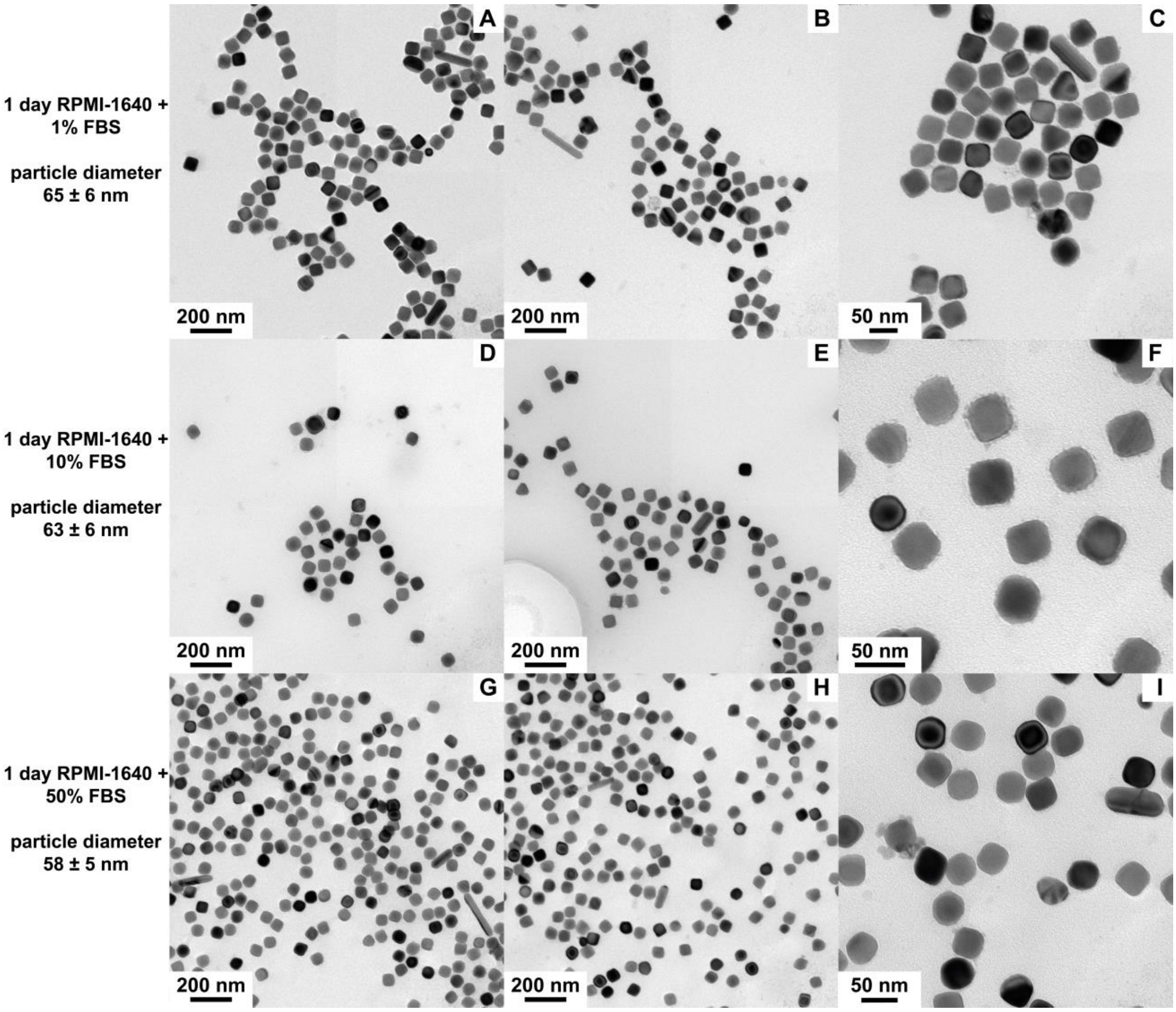

Supplementary Figure 13. Short incubation of silver nanocubes in cell culture media. TEM images of silver nanocubes after 24 hours incubation in RPMI-1640 supplemented with 1 % FBS **(A-C)**, 10 % FBS **(D-F)**, or 50 % FBS **(G-I)**. Scale bars are 200 nm **(A, B, D, E, G, H)** or 50 nm **(E, F, I).**

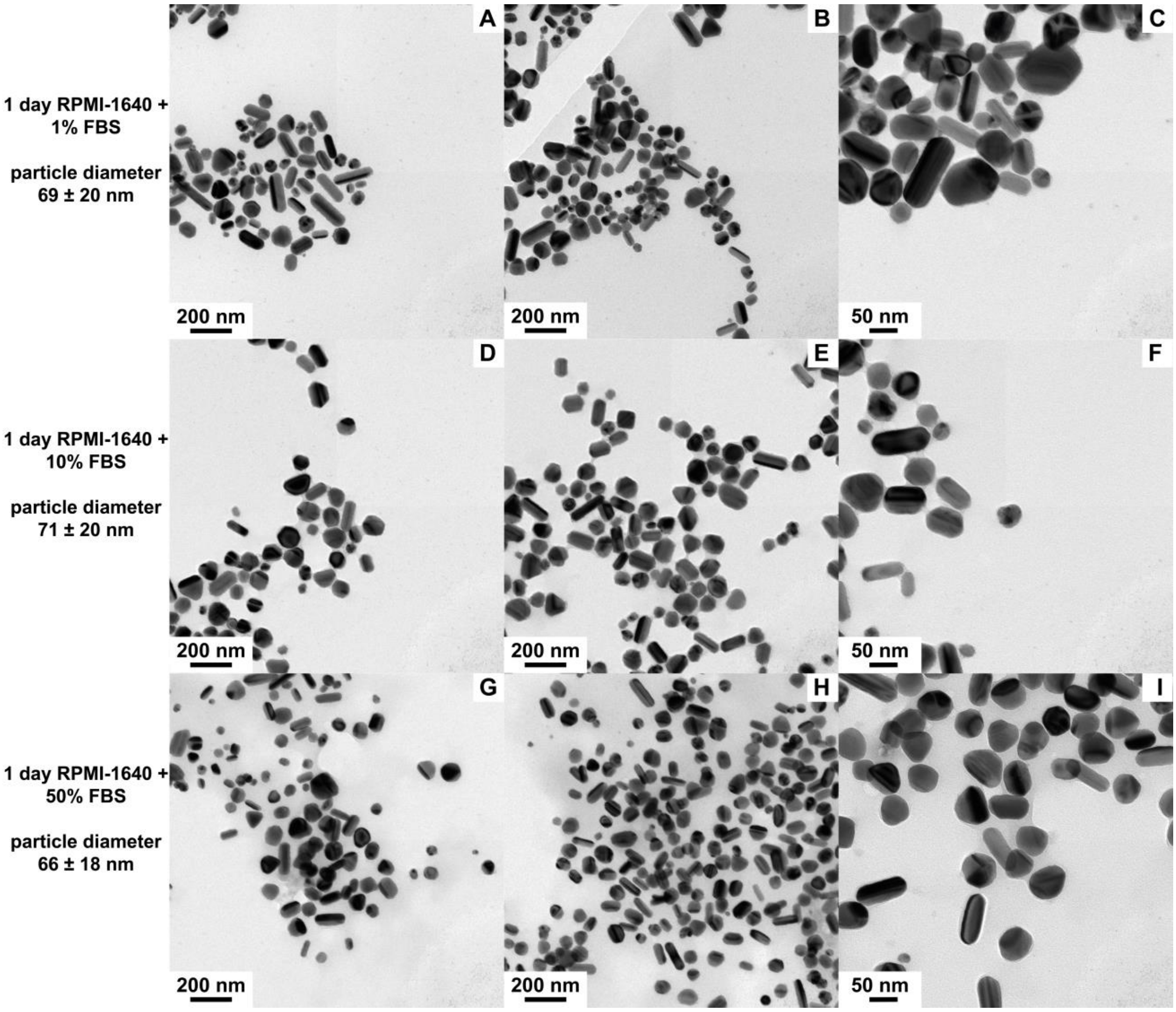

Supplementary Figure 14. Short incubation of quasi-spherical silver NPs in cell culture media TEM images of quasi-spherical silver nanoparticles after 24 hours incubation in RPMI-1640 supplemented with 1 % FBS **(A-C)**, 10 % FBS **(D-F)**, or 50 % FBS **(G-I)**. Scale bars are 200 nm **(A, B, D, E, G, H)** or 50 nm **(E, F, I).**

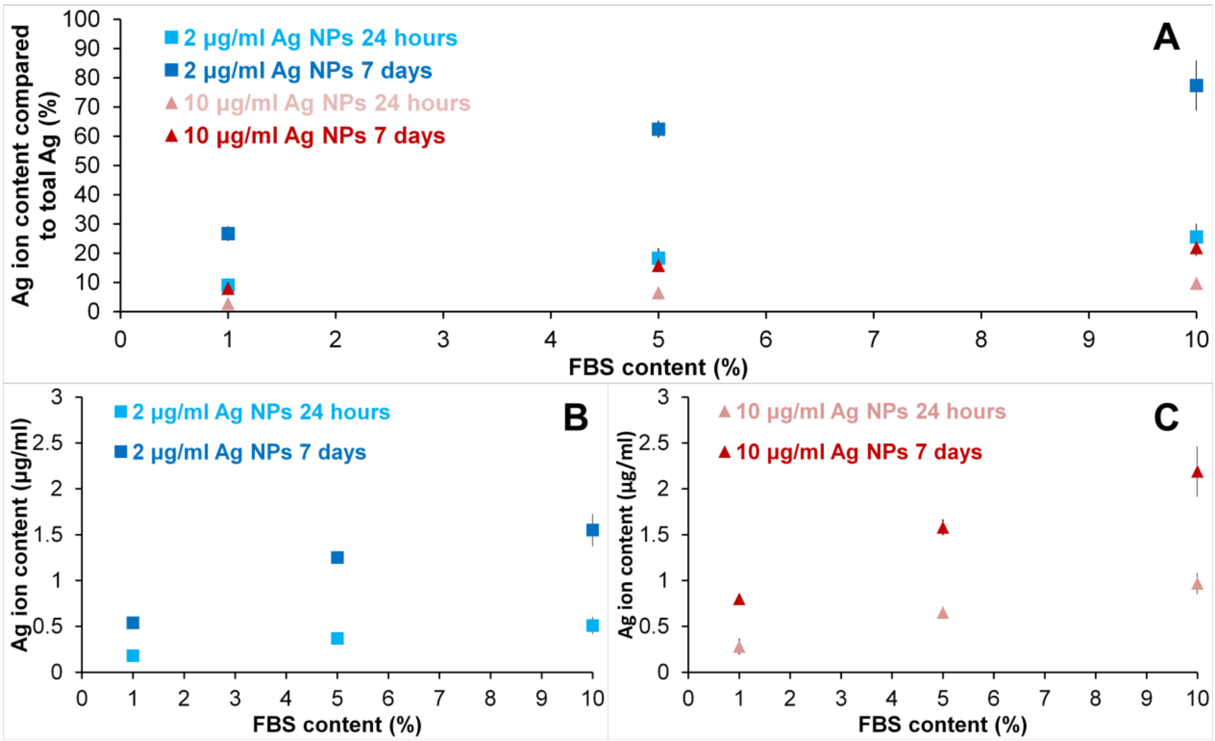

Supplementary Figure 15. Ion release of silver nanocubes. Ion release – expressed as percentage of total silver **(A)** or as µg ml^{-1} **(B, C)** – of silver nanocubes after 24 hours or 7 days incubation at an initial Ag concentration of 2 or 10 µg ml^{-1} in RPMI-1640 supplemented with either 1 % or 10 % FBS. Error bars are standard deviation.

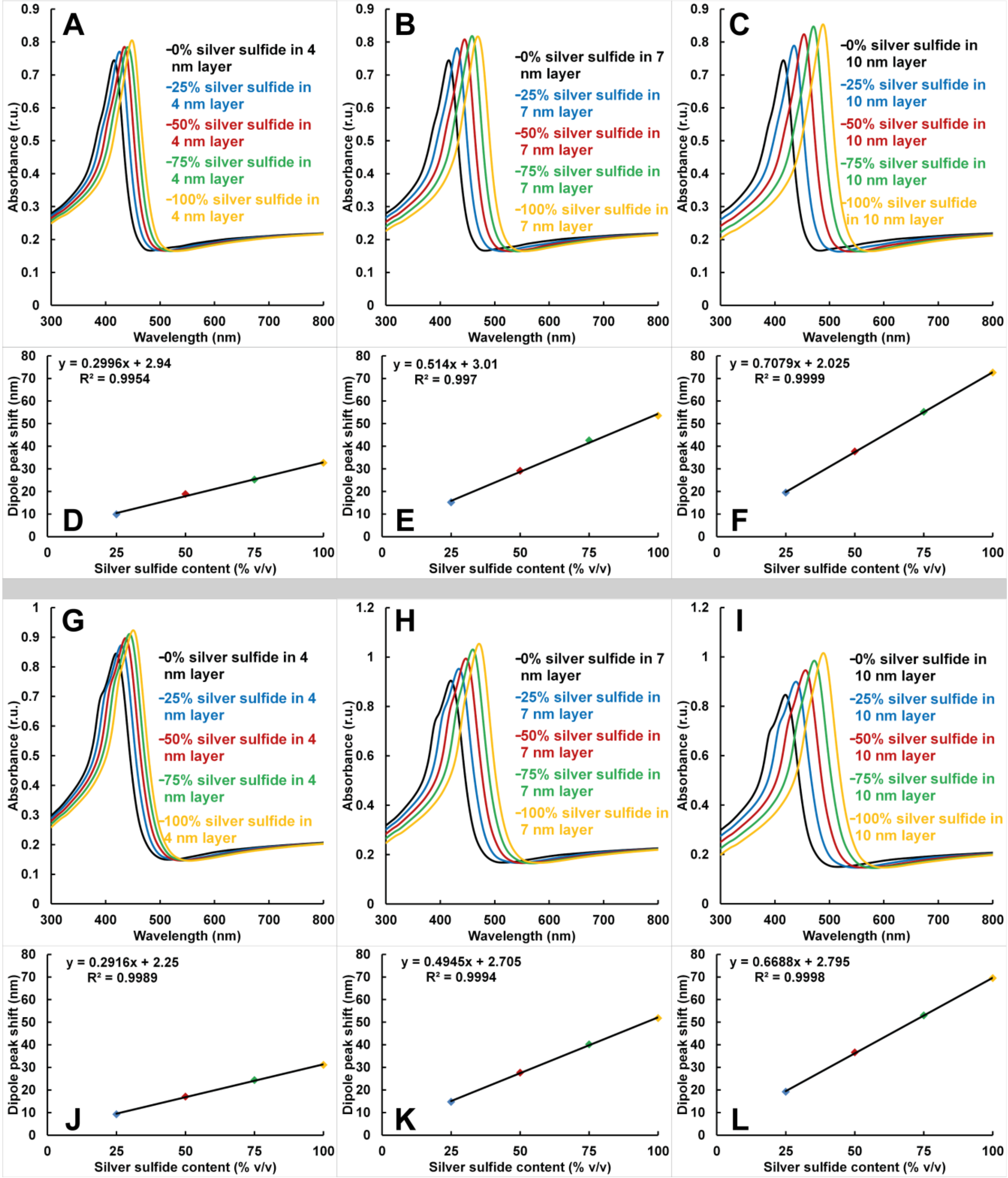

Supplementary Figure 16. FDTD simulations of sulphidated silver nanoparticles. FDTD simulations of 60 nm Ag NPs surrounded by a 4 nm **(A)**, 7 nm **(B)** and 10 nm **(C)** thick layer of Ag_2S and the respective calibration curves for peak-shift at maximum absorbance **(D-F)**. FDTD simulations of 70 nm Ag NPs surrounded by a 4 nm **(G)**, 7 nm **(H)** and 10 nm **(I)** thick layer of Ag_2S and the respective calibration curves for peak-shift at maximum absorbance **(J-L)**.

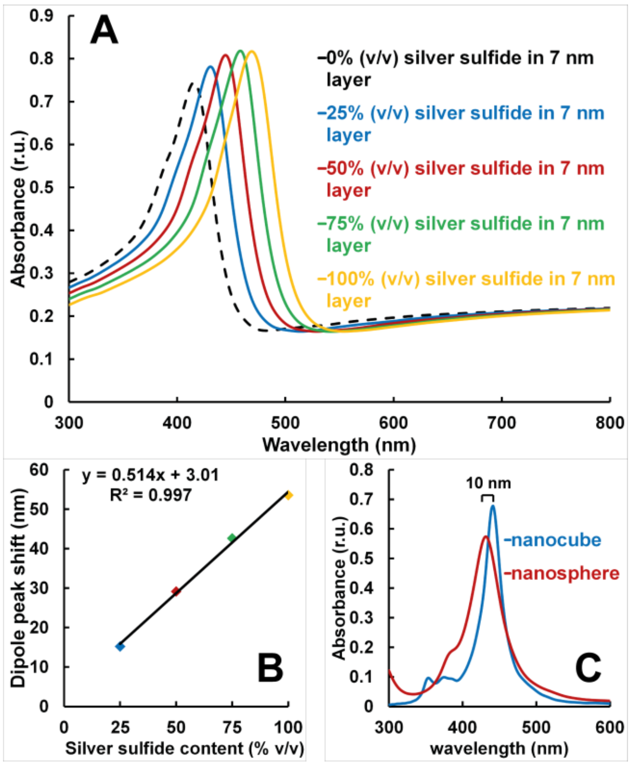

Supplementary Figure 17. FDTD simulations of cubic vs. quasi-spherical silver NPs FDTD simulations of 60 nm Ag NP surrounded by a 7 nm layer of Ag_2S with various degrees of occupancy **(A)** and the corresponding calibration curve **(B)**. FDTD simulated spectra of spherical and cubic silver nanoparticles of 60 nm diameter, in water **(C)**.

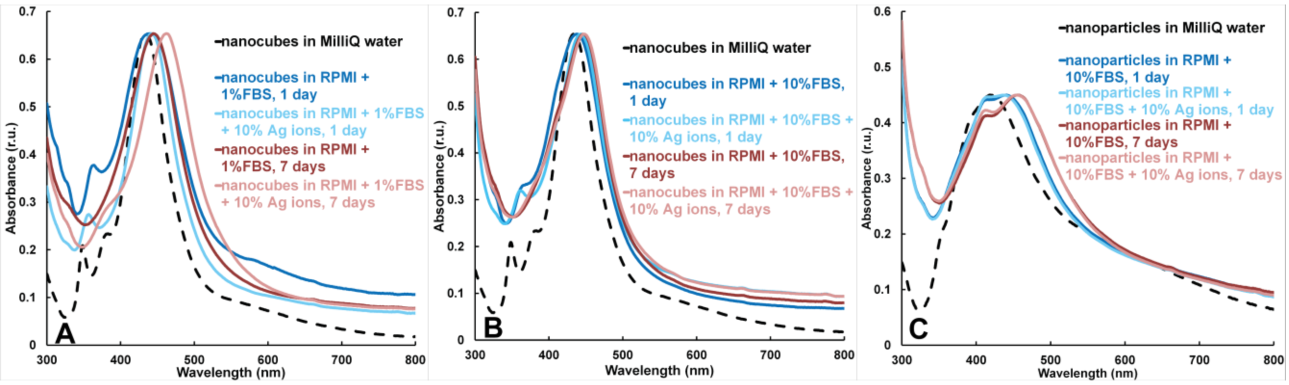

Supplementary Figure 18. Plasmonic response of silver nanocubes with added Ag^+. UV-vis spectra of silver nanocubes incubated in 1 % FBS with and without 10 % added Ag^+ **(A)**, 10 % FBS with and without added 10 % Ag^+ **(B)**, and quasi-spherical silver nanoparticles incubated in 10 % FBS with and without added 10 % Ag^+ **(C)**.

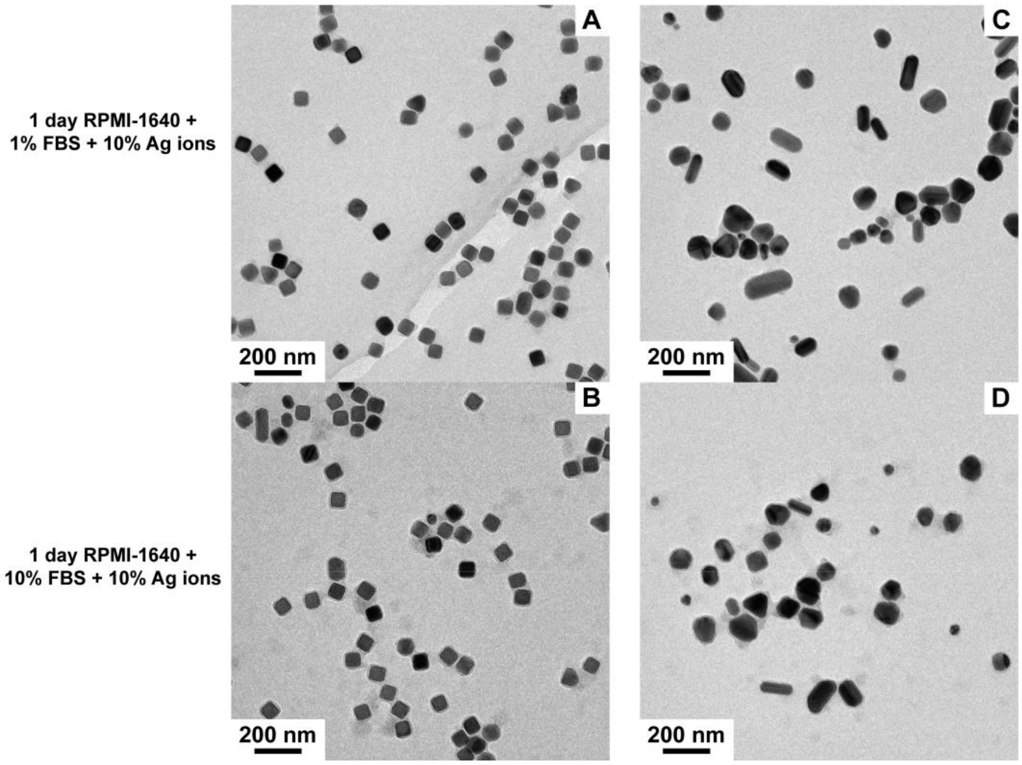

Supplementary Figure 19. Silver NPs after short-term incubation with extra Ag^+. TEM images of silver nanocubes after 24 hours incubation in RPMI-1640 with added extra 10 % Ag^+ and 1 % FBS **(A)** or 10 % FBS **(B)**; TEM images of quasi-spherical Ag NPs after 24 hours incubation in RPMI-1640 with added extra 10 % Ag^+ and 1 % FBS **(C)** or 10% FBS **(D)**. Scale bars are 200 nm.

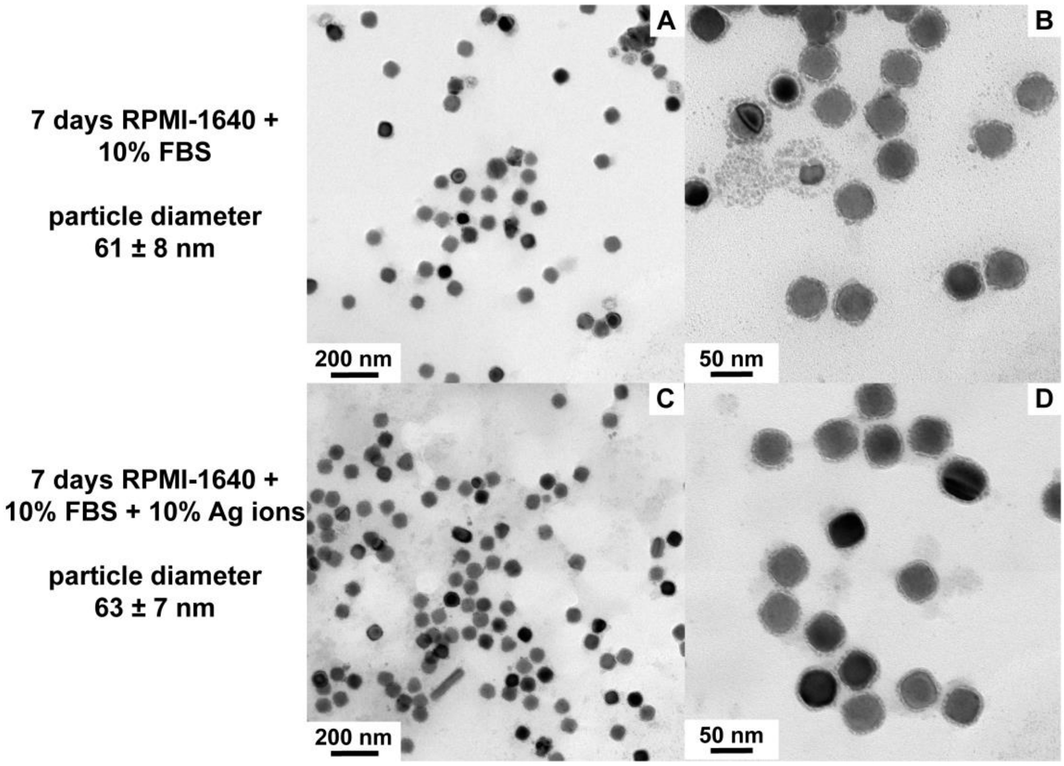

Supplementary Figure 20. Silver NPs after long incubation with extra Ag^{+}. TEM images of silver nanocubes after 7 days incubation in RPMI-1640 supplemented with 10 % FBS, without **(A, B)** and with **(C, D)** added extra 10 % Ag^{+} (by weight of the Ag NPs silver mass). Scale bars are 200 nm **(A, C)** or 50 nm **(B, D)**.

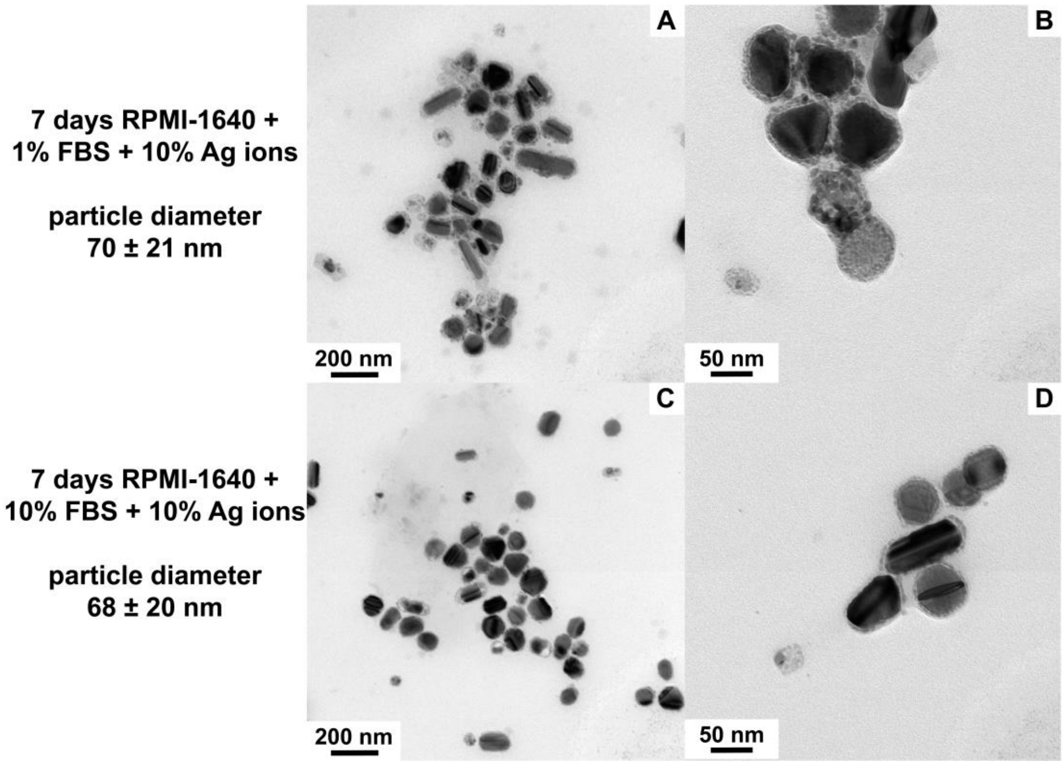

Supplementary Figure 21. Quasi-spherical silver NPs after long incubation with extra Ag^{+}. TEM images of quasi-spherical Ag NPs after 7 days incubation in RPMI-1640 cell culture medium supplemented with 10 % Ag ions (by weight of Ag NPs silver mass) and 1 % FBS **(A, B)** or 10 % FBS **(C, D)**. Scale bars are 200 nm **(A, C)** or 50 nm **(B, D)**.

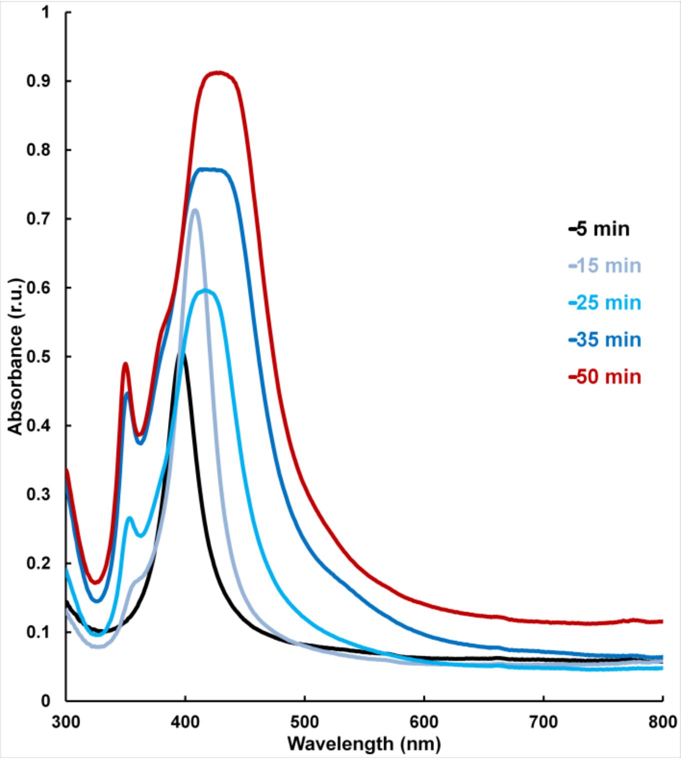

Supplementary Figure 22. Plasmonic response of silver nanocubes during synthesis. The synthesis of silver nanocubes is monitored collecting UV-vis spectra of samples obtained by adding a few drops of reaction mixture into 1 ml MilliQ water.

The appearance and increasing intensity of the localised surface plasmon resonance quadrupole peak around 350 nm is an indication of the formation and growth of silver nanocubes with more and more pronounced edges[1].

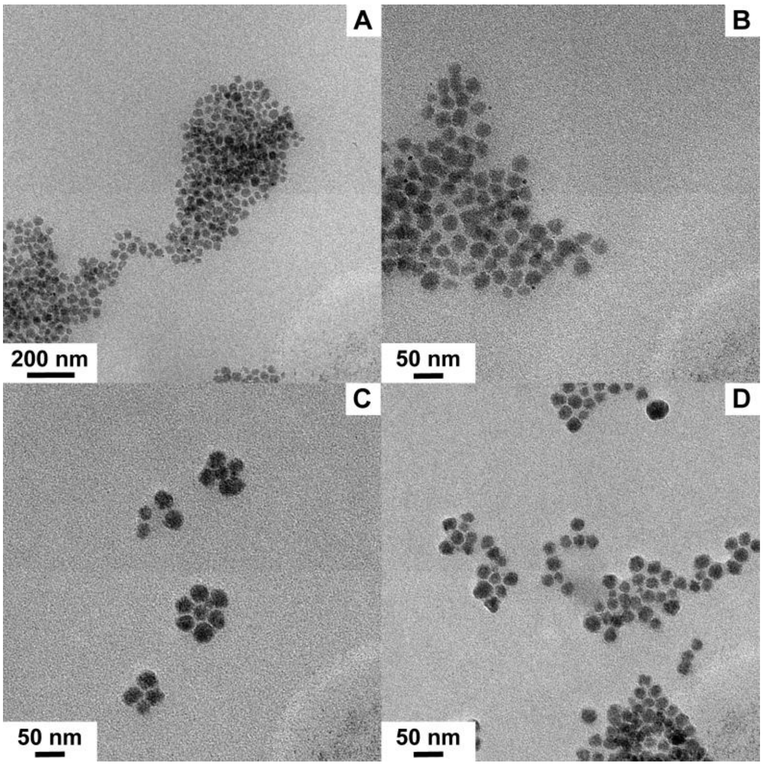

Supplementary Figure 23. Silica NPs in cell culture media with extra Ag^+. TEM images of silica nanoparticles after 7 days incubation in RPMI-1640 supplemented with 10 % Ag^+ (by weight of commonly used Ag NPs concentration) and 1 % FBS **(A, B)** or 10 % FBS **(C, D)**. Scale bars are 200 nm **(A)** or 50 nm **(B-D)**.

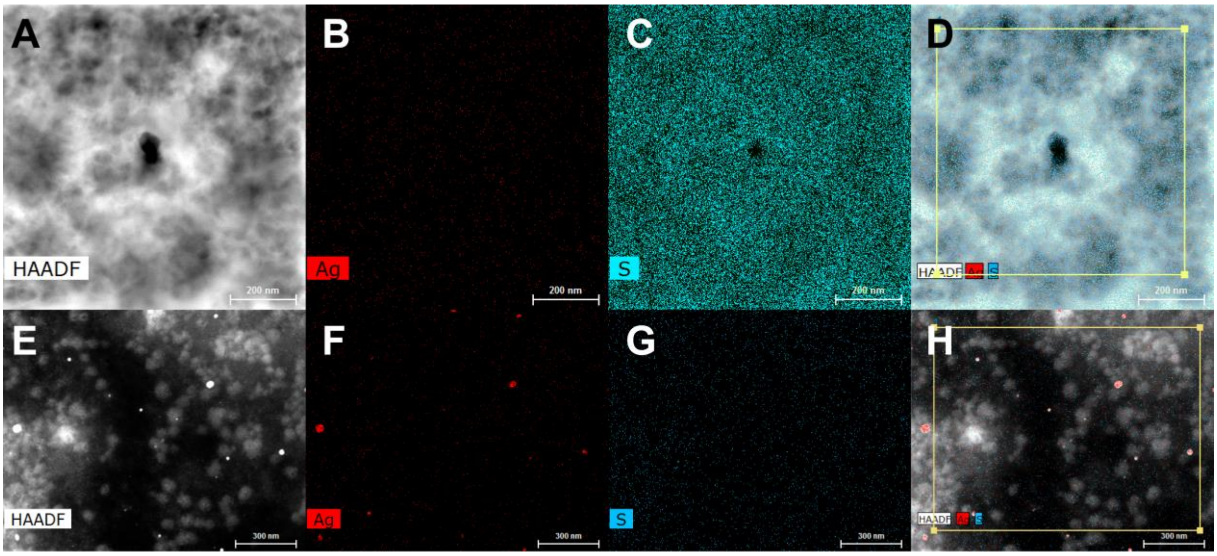

Supplementary Figure 24. Imaging of spiked and unspiked supernatants. TEM high-angle annular dark-field, silver elemental mapping, sulphur elemental mapping and overlaid image of un-spiked **(A-D)** and spiked **(E-H)** supernatant. Scale bars are 200 nm **(A-D)** or 300 nm **(E-H)**.

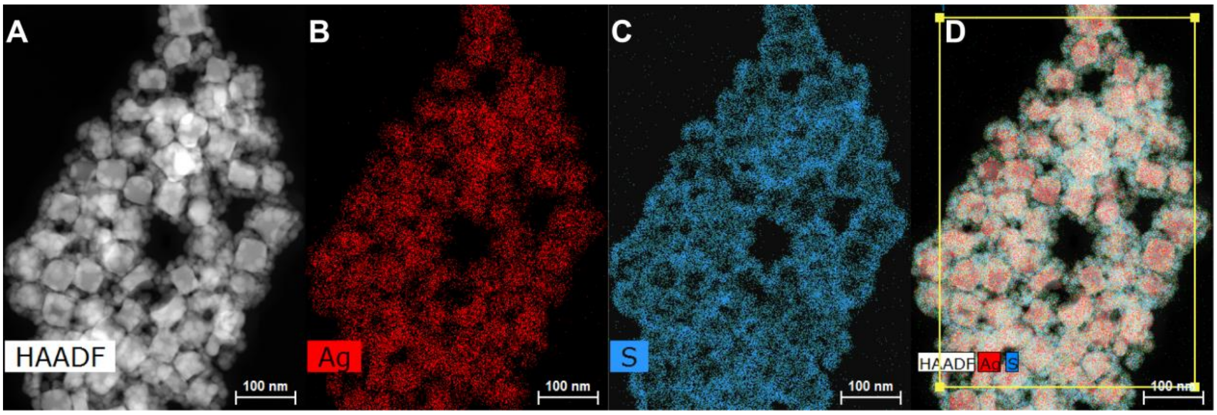

Supplementary Figure 25. Imaging of NPs in supplemented PBS. TEM high-angle annular dark-field **(A)**, silver **(B)**, sulphur **(C)** and overlapped **(D)** elemental mapping of Ag NPs after 24 hours incubation (2 µg ml^{-1}) in PBS supplemented with 20 µg ml^{-1} L-cysteine and 15 µg ml^{-1} L-methionine. Scale bars are 100 nm.

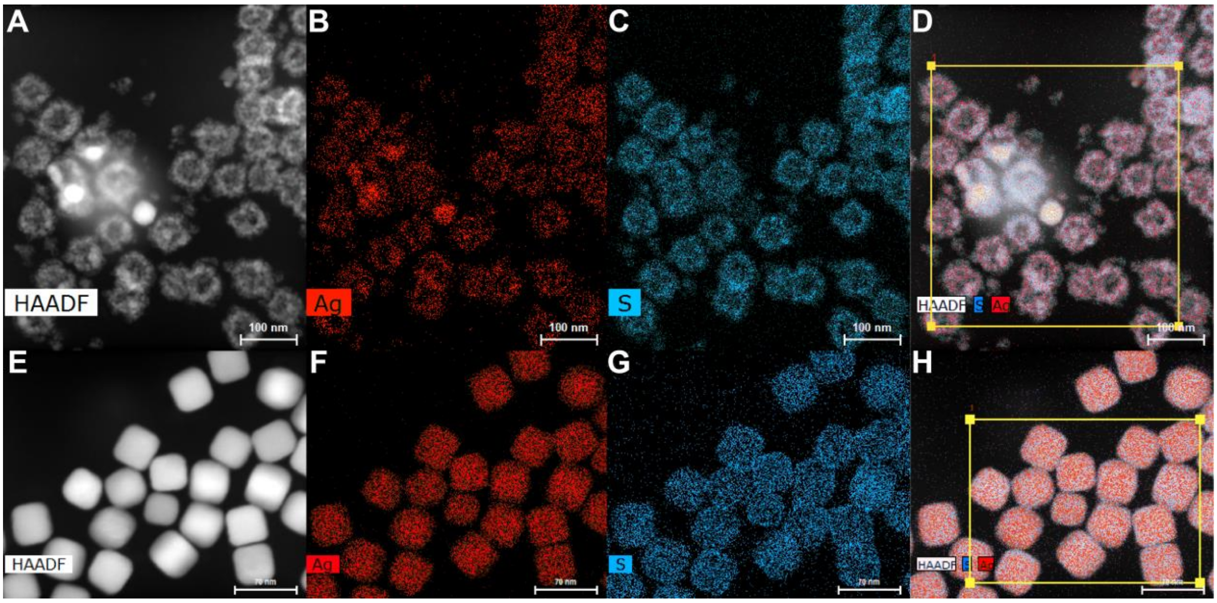

Supplementary Figure 26. Imaging of NPs incubated in cell culture media at various concentrations. TEM high-angular annular dark-field **(A, E)**, silver **(B, F)**, sulphur **(C, G)** and overlapped **(D, H)** elemental mapping of Ag NPs after 7 days incubation in RPMI-1640 supplemented with 1 % FBS, with an initial Ag concentration of 2 µg ml^{-1} and 100 µg ml^{-1} respectively. Scale bars are 100 nm **(A-D)** or 70 nm **(E-H)**.

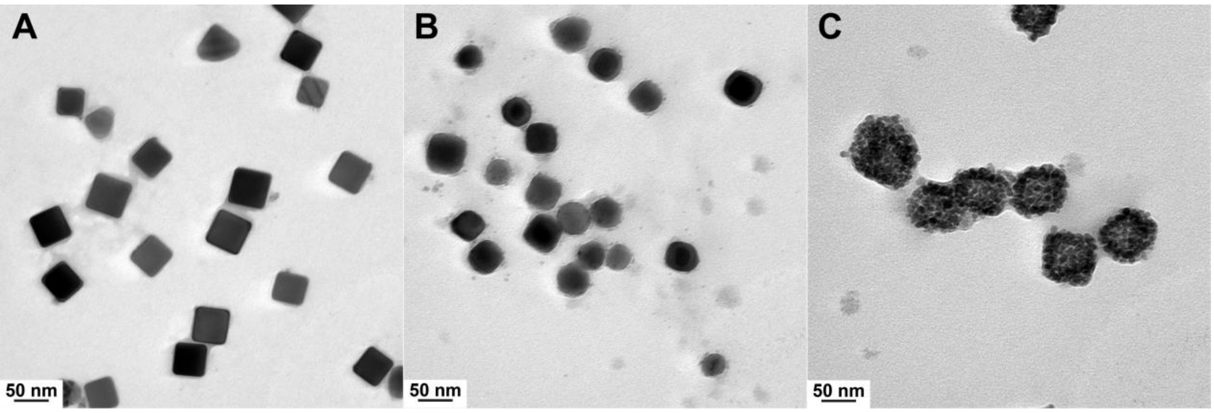

Supplementary Figure 27. Silver NPs used for toxicity experiments. TEM images of pristine **(A)**, partially sulphidated **(B)** and completely sulphidated **(C)** Ag NP samples used for toxicity cell studies. Partially and completely transformed nanoparticles were obtained by pre-incubating pristine Ag NPs in RPMI-1640 cell culture medium supplemented with 10 % FBS and 1 % FBS respectively. Scale bars are 50 nm.

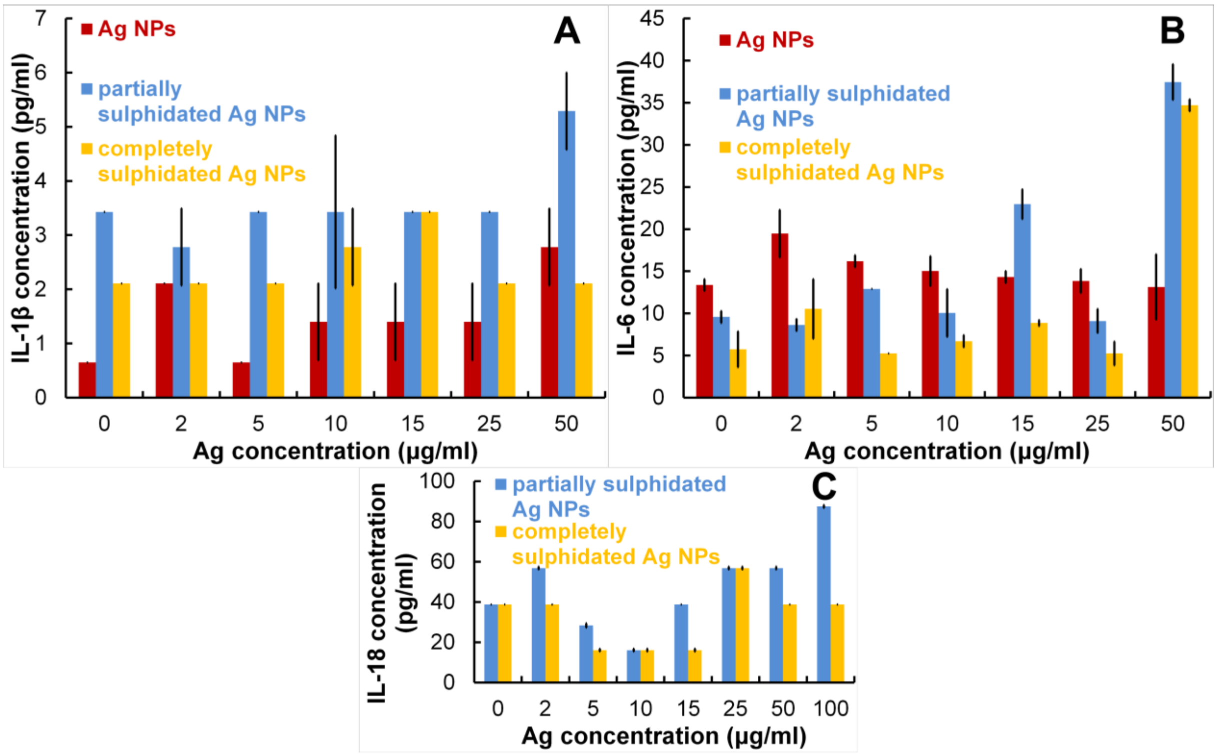

Supplementary Figure 28. Cytokine release profiles of J774 cells exposed to silver NPs. Interleukin-1β **(A)**, interleukin-6 **(B)** and interleukin-18 **(C)** release from J774 murine macrophage cells after 24 hours incubation with various concentrations (2, 5, 10, 15, 25 and 50 μg ml^{-1}) of pristine (red), partially (blue) and completely-sulphidated (orange) Ag NPs. Error bars are standard deviation.

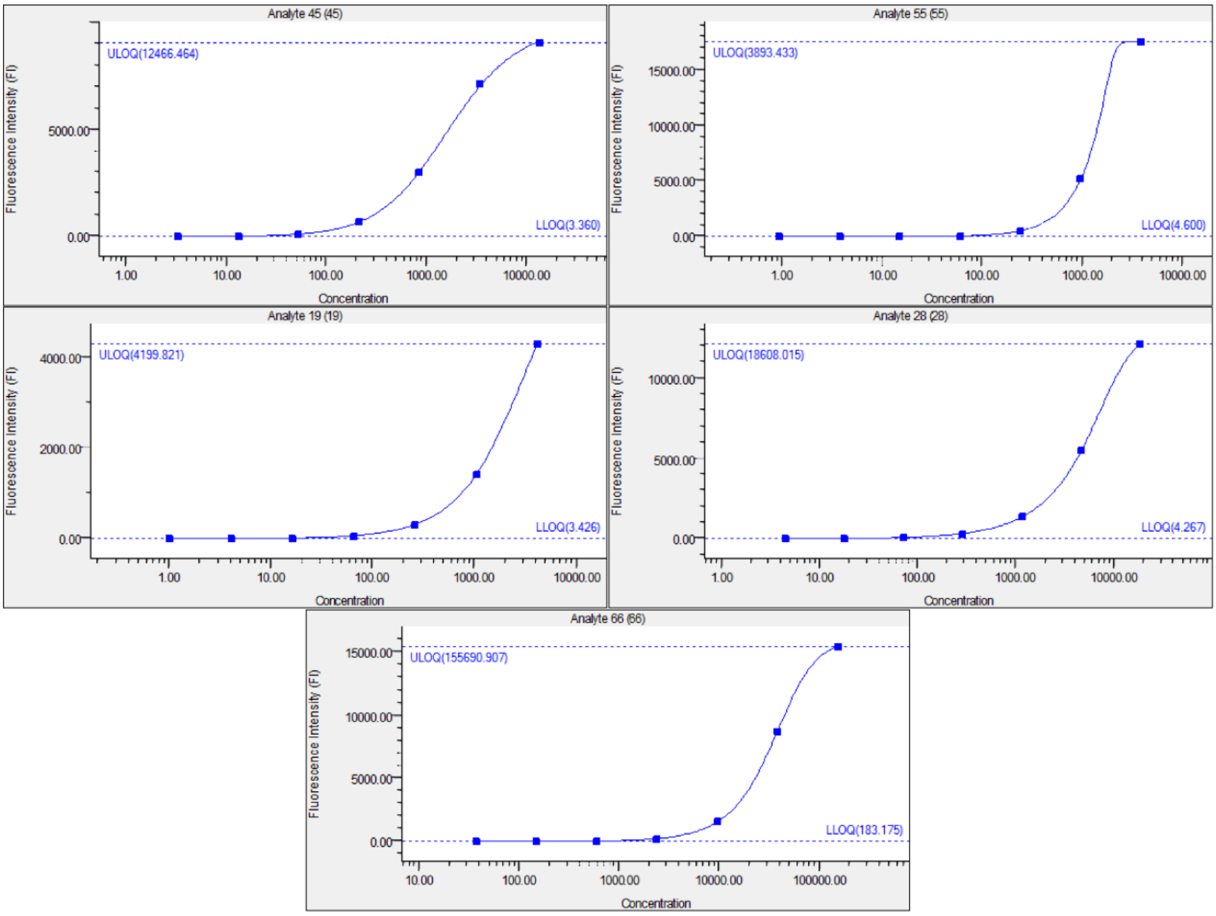

Supplementary Figure 29. Calibration curves of analysed cytokines. Analyte 45 = TNFα, Analyte 55 = MIP-2, Analyte 19 = IL-1β, Analyte 28 = IL-6 and Analyte 66 = IL-18, with all concentrations expressed in pg ml^{-1}.

**Protein**	Accession Number	*nanocubes 1% FBS 24 hours*	*nanocubes 10% FBS 24 hours*	*nanocubes 1% FBS 7 days*	*nanocubes 10% FBS 7 days*
Haemoglobin subunit α	P01966	17.7 ± 0.7	10.6 ± 0.7	9.1 ± 1.3	12.7 ± 0.9
Haemoglobin foetal subunit β	P02081	12.1 ± 0.5	7.6 ± 0.5	6.5 ± 1.1	-
Serum albumin	P02769	8.3 ± 0.5	5.4 ± 0.3	13.5 ± 0.9	8.1 ± 0.2
Haemoglobin subunit β	P02070	7.3 ± 0.2	4.4 ± 0.2	3.1 ± 0.2	5.1 ± 0.0
α-2-HS-glycoprotein	P12763	5.4 ± 0.8	7.4 ± 0.6	6.4 ± 0.7	10.3 ± 0.4
α-1-antiproteinase	P34955	3.5 ± 0.1	4.1 ± 0.2	4.6 ± 0.4	4.9 ± 0.2
Uncharacterised protein (C4A)	E1BH06	2.8 ± 0.0	9.2 ± 0.7	4.6 ± 0.5	4.6 ± 0.2
Uncharacterised protein (VTN)	Q3ZBS7	2.8 ± 0.3	2.4 ± 0.2	2.5 ± 0.2	2.0 ± 0.1
Plasminogen	E1B726	2.6 ± 0.2	1.4 ± 0.1	4.9 ± 0.4	1.1 ± 0.0
α-fetoprotein	Q3SZ57	1.4 ± 0.2	1.0 ± 0.1	1.6 ± 0.1	1.9 ± 0.1
α-2-macroglobulin	Q7SIH1	1.3 ± 0.1	0.8 ± 0.2	1.2 ± 0.1	1.4 ± 0.0
Complement C3	Q2UVX4	1.2 ± 0.1	1.1 ± 0.0	2.5 ± 0.5	1.0 ± 0.0
Protein S100-A8	P28782	1.2 ± 0.3	-	1.0 ± 07	0.9 ± 0.3
Inter-α-trypsin inhibitor heavy chain H2	F1MNW4	1.2 ± 0.2	0.7 ± 0.0	2.0 ± 0.1	5.3 ± 0.2
Uncharacterised protein (MYH9)	F1MQ37	1.1 ± 0.0	-	2.8 ± 0.3	-
Uncharacterised protein (IG like)	F1MLW8	1.1 ± 0.1	-	-	1.0 ± 0.1
Protein S-100-A12	P79105	1.0 ± 0.1	-	-	1.3 ± 0.3
Complement factor H	Q28085	0.8 ± 0.2	6.8 ± 0.7	3.1 ± 0.6	2.4 ± 0.2
Apolipoprotein A-I	P15497	0.8 ± 0.1	1.7 ± 0.1	-	1.6 ± 0.1
Actin, cytoplasmic 1	F1MRD0	0.8 ± 0.1	-	1.0 ± 0.2	0.8 ± 0.0
Protein S100-A9	E1BLI9	0.8 ± 0.3	-	1.2 ± 0.3	-
Apolipoprotein A-II	P81644	0.8 ± 0.0	1.0 ± 0.0	-	-
Apolipoprotein E	Q03247	0.8 ± 0.1	2.1 ± 0.1	1.1 ± 0.4	2.5 ± 0.1
Gelsolin	F1N1I6	0.7 ± 0.1	-	-	
Myosin-10	Q27991	0.6 ± 0.5	-	-	
Uncharacterised	E1BNR0	-	3.4 ± 0.4	1.1 ± 0.2	1.9 ± 0.2

protein (APOB)					
C4b-binding protein alpha chain	Q28065	-	2.3 ± 0.4	-	-
C-X-C motif chemokine	F1MD83	-	1.6 ± 0.2	-	-
Sulfhydryl oxidase	F1MM32	-	1.5 ± 0.2	-	-
Fibronectin	P07589	-	1.4 ± 0.1	-	-
Tetranectin	Q2KIS7	-	1.2 ± 0.4	-	-
Coagulation factor XIII A chain	F1MW44	-	1.1 ± 0.7	-	-
Uncharacterised protein (SERPING1)	E1BMJ0	-	1.0 ± 0.0	-	-
Complement factor B	P81187	-	-	1.1 ± 0.1	-
Antithrombin-III	F1MSZ6	-	-	1.0 ± 0.4	-
Inter-α-trypsin inhibitor heavy chain H4	F1MMD7	-	-	1.0 ± 0.2	1.1 ± 0.0
Kininogen-1	P01044	-	-	1.0 ± 0.1	-
Kininogen-2	P01045	-	-	1.0 ± 0.1	-
Fetuin-B	Q58D62	-	-	-	0.9 ± 0.3
Transthyretin	O46375	-	-	-	0.9 ± 0.0
Protein AMBP	P00978	-	-	-	0.8 ± 0.2

Supplementary Table 1. Silver nanocubes main hard corona components. Major components of silver nanocube serum protein hard coronas, with proteins highlighted in yellow being common to all coronas and making up between 55 and 70 % of the hard corona.

**Protein**	Accession Number	*nanoparticles 1% FBS 24 hours*	*nanoparticles 10% FBS 24 hours*	*nanoparticles 1% FBS 7 days*	*nanoparticles 10% FBS 7 days*
Haemoglobin subunit α	P01966	16.0 ± 1.6	11.3 ± 0.4	8.7 ± 2.4	12.2 ± 0.7
Haemoglobin foetal subunit β	P02081	8.8 ± 2.3	7.2 ± 0.1	4.7 ± 0.6	7.8 ± 0.6
Serum albumin	P02769	8.2 ± 1.3	5.1 ± 0.2	12.3 ± 0.7	9.9 ± 0.2
α-2-HS-glycoprotein	P12763	7.6 ± 1.2	5.4 ± 0.7	7.8 ± 2.0	9.1 ± 0.4
Haemoglobin subunit β	P02070	5.3 ± 1.6	4.4 ± 0.0	3.8 ± 0.9	4.5 ± 1.1
α-1-antiproteinase	P34955	4.5 ± 0.3	3.3 ± 0.0	4.1 ± 0.1	5.0 ± 0.2
Plasminogen	E1B726	3.5 ± 0.2	1.3 ± 0.1	4.2 ± 0.1	1.3 ± 0.0
Uncharacterised protein (C4A)	E1BH06	3.5 ± 0.3	8.0 ± 0.5	3.5 ± 0.4	4.2 ± 0.1
Protein S100-A8	P28782	2.0 ± 0.5	-	2.2 ± 0.2	0.9 ± 0.0
Complement C3	Q2UVX4	1.9 ± 0.2	0.9 ± 0.1	2.4 ± 0.2	0.9 ± 0.0
Uncharacterised protein (VTN)	Q3ZBS7	1.8 ± 0.2	3.3 ± 0.1	3.1 ± 0.4	2.1 ± 0.1
Inter-α-trypsin inhibitor heavy chain H2	F1MNW4	1.7 ± 0.1	-	1.2 ± 0.2	6.2 ± 0.3
α-2-macroglobulin	Q7SIH1	1.7 ± 0.1	0.8 ± 0.0	1.1 ± 0.1	1.4 ± 0.0
Uncharacterised protein (MYH9)	F1MQ37	1.5 ± 0.2	-	2.6 ± 0.2	-
Uncharacterised protein (IG like)	F1MLW8	1.5 ± 0.1	-	0.8 ± 0.1	1.0 ± 0.0
Complement factor H	Q28085	1.4 ± 0.2	7.6 ± 0.1	1.9 ± 0.6	2.3 ± 0.1
Protein S100-A9	E1BLI9	1.4 ± 0.3	1.4 ± 0.0	1.2 ± 0.6	-
Protein S-100-A12	P79105	1.3 ± 0.2	-	0.9 ± 0.4	0.8 ± 0.1
α-fetoprotein	Q3SZ57	1.3 ± 0.0	1.0 ± 0.1	1.4 ± 0.1	2.2 ± 0.0
Apolipoprotein A-I	P15497	0.9 ± 0.1	1.7 ± 0.0	-	1.3 ± 0.0
Inter-α-trypsin inhibitor heavy chain H4	F1MMD7	0.9 ± 0.1	-	0.8 ± 0.1	1.0 ± 0.0
Actin, cytoplasmic 1	F1MRD0	0.8 ± 0.0	-	0.8 ± 0.1	0.8 ± 0.0
Apolipoprotein E	Q03247	0.8 ± 0.4	1.5 ± 0.0	0.7 ± 0.0	1.8 ± 0.0
Transthyretin	O46375	0.8 ± 0.1	-	-	-
Uncharacterised protein (IGLL1)	F1MLW7	0.8 ± 0.2	-	-	-

C4b-binding protein alpha chain	Q28065	-	1.8 ± 0.0	-	-
Uncharacterised protein (APOB)	E1BNR0	-	1.7 ± 0.1	-	1.4 ± 0.1
Coagulation factor XIII A chain	F1MW44	-	1.6 ± 0.0	-	-
Apolipoprotein A-II	P81644	-	1.4 ± 0.3	-	1.7 ± 0.1
Fibronectin	P07589	-	1.4 ± 0.0	-	-
C-X-C motif chemokine	F1MD83	-	1.4 ± 0.1	-	-
Sulfhydryl oxidase	F1MM32	-	1.2 ± 0.1	-	-
Tetranectin	Q2KIS7	-	1.1 ± 0.3	-	-
C1QTNF3 protein	A7MB82	-	0.9 ± 0.0	-	-
Gelsolin	F1N1I6	-	-	1.0 ± 0.1	-
Antithrombin-III	F1MSZ6	-	-	1.2 ± 0.1	-
Prothrombine	P00735	-	-	0.6 ± 0.1	-
Protein AMBP	P00978	-	-	-	1.1 ± 0.1
Fetuin-B	Q58D62	-	-	-	0.9 ± 0.0

Supplementary Table 2. Quasi-spherical silver NPs main hard corona components. Major components of silver quasi-spherical nanoparticles serum protein hard coronas, with proteins highlighted in yellow being common to all coronas and making up between 50 and 65 % of the hard corona.

Protein	Accession number	*C-1 24 h*	*C-10 24 h*	*C-1 7 d*	*C-10 7 d*	*S-1 24 h*	*S-10 24 h*	*S-1 7 d*	*S-10 7 d*
Serum albumin	P02769	x	x	x	x	x	x	x	x
Uncharacterized protein	E1BH06	x	x	x	x	x	x	x	x
Hemoglobin fetal subunit beta	P02081	x	x	x	x	x	x	x	x
Complement C3	Q2UVX4	x	x	x	x	x	x	x	x
Uncharacterized protein	F1MQ37	x	x	x	x	x	x	x	x
Uncharacterized protein (Fragment)	F1MVK1	x	x	x	x	x	x	x	x
Hemoglobin subunit alpha	P01966	x	x	x	x	x	x	x	x
Uncharacterized protein	Q3ZBS7	x	x	x	x	x	x	x	x
Alpha-2-macroglobulin	Q7SIH1	x	x	x	x	x	x	x	x
Alpha-2-HS-glycoprotein	P12763	x	x	x	x	x	x	x	x
Hemoglobin subunit beta	P02070	x	x	x	x	x	x	x	x
Plasminogen	E1B726	x	x	x	x	x	x	x	x
Alpha-1-antiproteinase	P34955	x	x	x	x	x	x	x	x
Complement C4 (Fragments)	P01030	x	x	x	x	x	x	x	x
Prothrombin	P00735	x	x	x	x	x	x	x	x
Apolipoprotein A-I	P15497	x	x	x	x	x	x	x	x
Angiotensinogen	Q3SZH5	x	x	x	x	x	x	x	x
Pigment epithelium-derived factor	Q95121	x	x	x	x	x	x	x	x
Inter-alpha-trypsin inhibitor heavy chain H4	F1MMD7	x	x	x	x	x	x	x	x
Complement factor H	Q28085	x	x	x	x	x	x	x	x
Alpha-fetoprotein	Q3SZ57	x	x	x	x	x	x	x	x
Glyceraldehyde-3-phosphate dehydrogenase	P10096	x	x	x	x	x	x	x	x
Uncharacterized protein	F1MLW7	x	x	x		x	x		x
Actin, cytoplasmic 1	P60712	x	x	x	x	x	x	x	x
Coagulation factor V	Q28107	x	x	x	x	x	x	x	x
Inter-alpha-trypsin inhibitor heavy chain H2	F1MNW4	x	x	x	x	x	x	x	x
Clusterin	P17697	x	x	x	x	x	x	x	x
Alpha-1B-glycoprotein	Q2KJF1	x	x	x	x	x	x	x	x

Apolipoprotein A-II	P81644	x	x	x	x	x	x	x	x
Tubulin alpha-1B chain	P81947	x		x		x		x	
Uncharacterized protein	F1MRZ8	x	x	x	x	x	x	x	x
Complement factor B	P81187	x	x	x	x	x	x	x	x
Uncharacterized protein	F1MLW8	x	x	x	x	x	x	x	x
Antithrombin-III	F1MSZ6	x	x	x	x	x	x	x	x
Apolipoprotein E	Q03247	x	x	x	x	x	x	x	x
Thrombospondin-1	F1N3A1	x	x	x	x	x	x	x	x
Inter-alpha-trypsin inhibitor heavy chain H3	P56652	x	x	x	x	x	x	x	x
Lactotransferrin	P24627	x	x	x	x	x	x	x	x
Gelsolin	Q3SX14	x	x	x	x	x	x	x	x
Sulfhydryl oxidase	F1MM32	x	x	x	x	x	x	x	x
Uncharacterized protein (Fragment)	F1MDH3	x		x		x		x	
Peptidoglycan recognition protein 1	Q8SPP7	x	x	x	x	x	x	x	x
Transthyretin	O46375	x	x	x	x	x	x	x	x
Keratin, type II cytoskeletal 75	Q08D91	x			x			x	
Protein S100-A8	P28782	x	x	x	x	x	x	x	x
Uncharacterized protein (Fragment)	G5E513	x	x	x	x	x	x	x	x
Alpha-enolase	Q9XSJ4	x	x	x	x	x	x	x	x
Coagulation factor X	P00743	x	x	x	x	x	x	x	x
Uncharacterized protein	G3N0V2	x	x		x	x	x	x	x
Uncharacterized protein	E1BNR0	x	x	x	x	x	x	x	x
Keratin, type I cytoskeletal 14	F1MC11	x	x		x	x	x	x	x
SERPIND1 protein	A6QPP2	x	x	x	x	x	x	x	x
Fetuin-B	Q58D62	x	x	x	x	x	x	x	x
Heat shock 70 kDa protein 1A	Q27975	x		x		x		x	
C-reactive protein	C4T8B4	x	x	x	x	x	x	x	x
Fibrinogen gamma-B chain	F1MGU7	x	x	x	x	x	x	x	x
Metallothionein-1A	P67983	x	x	x	x	x	x	x	x
Cartilage oligomeric matrix protein	P35445	x	x	x	x	x	x	x	x

Alpha-2-antiplasmin	P28800	x	x	x	x	x	x	x	x
Uncharacterized protein	G3MWV5	x	x	x	x	x	x	x	x
Uncharacterized protein	F1MI18	x	x	x	x	x	x	x	x
Heat shock protein HSP 90-alpha	Q76LV2	x	x	x		x		x	
Secreted phosphoprotein 24	Q27967	x	x	x	x	x	x	x	x
Spleen trypsin inhibitor I	P04815	x	x	x	x	x	x	x	x
Uncharacterized protein (Fragment)	G3N0V0	x	x	x	x	x		x	x
Tetranectin	Q2KIS7	x	x	x	x	x	x	x	x
Lumican	Q05443	x	x	x	x	x	x	x	x
Thrombospondin-4	Q3SWW8	x	x	x	x	x	x	x	x
Heat shock cognate 71 kDa protein	P19120	x							
Vitamin K-dependent protein C (Fragment)	P00745	x	x	x	x	x		x	
Ras-related protein Rap-1A	P62833	x	x	x	x	x	x	x	
Thyroxine-binding globulin	Q9TT36	x	x	x	x	x	x	x	x
Protein S100-A9	E1BLI9	x	x	x	x	x	x	x	x
Vitamin D-binding protein	Q3MHN5	x	x	x	x	x	x	x	x
ALDOA protein	A6QLL8	x	x	x	x	x	x	x	x
Uncharacterized protein	F1N169	x		x		x		x	
Fibrinogen alpha chain	A5PJE3	x	x	x	x	x	x	x	x
Leucine-rich alpha-2-glycoprotein 1	Q2KIF2	x	x	x	x	x	x	x	x
Inter-alpha-trypsin inhibitor heavy chain H1	F1MMP5	x	x	x	x	x	x	x	x
Keratin 31	Q148I8	x						x	
Beta-2-microglobulin	P01888	x	x	x		x	x	x	
C-X-C motif chemokine	F1MD83	x	x	x	x	x	x	x	x
Flavin reductase (NADPH)	P52556	x				x		x	
Uncharacterized protein	E1BJK2	x		x		x		x	
Uncharacterized protein	A0A0A0MP90	x	x	x	x		x	x	x
Adenosylhomocysteinase	Q3MHL4	x	x	x		x	x	x	
Protein S100-A12	P79105	x	x	x	x	x	x	x	x

Beta-2-glycoprotein 1	P17690	x	x	x	x	x	x	x	x
Hemopexin	Q3SZV7	x	x	x	x	x	x	x	x
Fibulin-1	F1MYN5	x	x	x	x	x	x	x	x
Fibrinogen beta chain	F1MAV0	x	x	x	x	x	x	x	x
Uncharacterized protein	G3N0Q8	x	x	x	x	x	x	x	x
CLEC11A protein	A5D7L1	x	x	x		x		x	
Uncharacterized protein	E1BI98	x			x	x	x	x	x
Complement factor properdin	Q17QC8	x	x	x	x	x	x	x	x
Serum amyloid P-component	Q3T004	x	x	x	x	x	x	x	x
Apolipoprotein A-IV	F1N3Q7	x	x	x		x	x	x	
Complement component C7	F1N045	x	x	x	x	x	x	x	x
Leukocyte cell-derived chemotaxin-2	O62644	x	x	x	x		x	x	x
Uncharacterized protein (Fragment)	F1MJZ4	x		x		x		x	
Alpha-amylase	F1MJQ3	x		x		x		x	
Complement C5a anaphylatoxin	F1MY85	x	x	x	x		x	x	x
Plasma kallikrein	Q2KJ63	x	x	x	x	x	x	x	x
Carboxypeptidase N catalytic chain	Q2KJ83	x	x	x		x	x	x	
L-lactate dehydrogenase B chain	Q5E9B1	x		x				x	
Uncharacterized protein (Fragment)	G5E604	x	x	x	x	x	x	x	x
Histone H2B	E1B8G9	x	x	x	x	x	x	x	x
Serpin A3-7	A0A0A0MP92	x	x		x	x	x		x
Coagulation factor IX	F1MFL4	x	x	x		x		x	
Hyaluronan-binding protein 2	Q5E9Z2	x	x	x	x	x	x	x	x
Matrix Gla protein	P07507	x	x				x	x	
14-3-3 protein zeta/delta	P63103	x							
Myosin light polypeptide 6	P60661	x	x	x		x	x	x	
Ribonuclease 4	Q58DP6	x	x	x	x	x	x	x	x
Acidic mammalian chitinase	Q95M17	x	x	x	x	x	x	x	x
Coagulation factor XIII, B	Q2TBQ1	x	x	x	x	x	x	x	

polypeptide									
Histidine-rich glycoprotein	F1MKS5	x	x	x	x	x	x	x	x
Uncharacterized protein (Fragment)	G3N2D7	x	x	x	x	x	x	x	x
Corticosteroid-binding globulin	E1BF81	x	x	x	x		x	x	x
Uncharacterized protein	F1MW79	x	x	x		x	x	x	x
Complement component C9	Q3MHN2	x	x	x	x	x	x	x	
Uncharacterized protein	F1N789	x							
Uncharacterized protein	F1MHR4	x	x	x		x	x	x	
Uncharacterized protein	F1MX86	x							
Uncharacterized protein (Fragment)	G5E5T5	x	x	x		x	x	x	
Fibronectin	P07589	x	x	x	x	x	x	x	x
Integrin beta	F1MTN1	x							
Uncharacterized protein	F1MR86	x		x		x		x	
Vitamin K-dependent protein Z	P00744	x	x	x	x	x	x	x	x
Chloride intracellular channel protein 1	Q5E9B7	x							
Collagen alpha-1(X) chain	P23206	x	x	x		x	x		
Triosephosphate isomerase	Q5E956	x				x		x	
Procollagen C-endopeptidase enhancer	Q2HJB6	x							
Vitamin K-dependent protein S	P07224	x	x	x		x	x	x	
C1QTNF3 protein	A7MB82	x	x		x		x	x	x
Carboxypeptidase B2	Q2KIG3	x	x	x	x	x	x	x	x
GTP-binding nuclear protein Ran	Q3T054	x		x		x		x	
Myocilin	Q9XTA3	x						x	
Annexin A6	P79134	x							
Collectin-11	Q17QH6	x	x	x	x	x	x	x	x
Proteasome subunit beta type-4	Q3T108	x		x		x	x	x	

Collagen alpha-1(II) chain	F1MSR8	x	x	x	x		x	x	x
Transketolase	Q6B855	x							
Profilin	E1BHJ0	x		x		x	x	x	
Asporin	Q3ZBN5	x	x	x		x	x	x	
Collagen triple helix repeat containing 1	A2VDY0	x							
Uncharacterized protein	G3N0S9	x	x		x		x	x	x
Cathepsin Z	P05689	x				x			
Guanine nucleotide-binding protein G(I)/G(S)/G(T) subunit beta-1	P62871	x							
Insulin-like growth factor-binding protein 5	Q05717	x					x	x	
Uncharacterized protein	E1BB91	x	x				x	x	
Peroxiredoxin-2	Q9BGI3	x							
Ras-related protein Rab-11A	F2Z4D5	x							
Coagulation factor XI	F1MUT4	x	x	x				x	
Regucalcin	Q9TTJ5	x			x	x		x	x
Histone H3	E1BGN3	x	x	x			x	x	
C1QC protein (Fragment)	Q1RMH5	x							
C4b-binding protein alpha chain	Q28065	x	x	x	x	x	x	x	x
Dihydrodiol dehydrogenase 3	P52898	x		x				x	
Adenylyl cyclase-associated protein 1	Q3SYV4	x				x			
Angiogenin-1	P10152	x	x	x	x		x	x	x
ECM1 protein	A5PJT7	x	x	x	x		x	x	x
SERPINA10 protein	A5PJ69	x		x	x	x		x	
Prostaglandin E synthase 3	Q3ZBF7	x							
Uncharacterized protein	E1BMJ0	x	x	x	x	x	x	x	x
Complement factor D	Q3T0A3	x							
Macrophage migration inhibitory factor	P80177	x							
14-3-3 protein beta/alpha	P68250	x		x					
Aldose reductase	P16116	x				x		x	
Alpha-1-acid glycoprotein	Q3SZR3	x						x	

Complement C1s subcomponent	Q0VCX1	x		x		x		x	
Connective tissue growth factor	O18739	x	x	x	x		x	x	x
WD repeat-containing protein 1	F1MTP5	x				x		x	
60S ribosomal protein L35	Q3MHM7	x							
Uncharacterized protein	F1MVB0	x		x		x			
Coagulation factor XII	F1MTT3	x	x	x	x		x	x	
Coagulation factor XIII A chain	F1MW44	x	x	x	x		x	x	x
Uncharacterized protein	F1MZX6	x	x	x		x		x	
Heat shock 70kD protein binding protein	A7E3S8	x				x		x	
Uncharacterized protein	E1BMK2	x		x				x	
Fermitin family homolog 3	Q32LP0	x		x		x		x	
Uncharacterized protein	F1MYX2	x	x	x	x		x	x	x
Uncharacterized protein	E1BCJ2	x	x	x			x		
Alcohol dehydrogenase [NADP(+)]	Q3ZCJ2	x						x	
Mimecan	P19879	x		x		x		x	
Uncharacterized protein	G3MYZ3	x	x	x	x			x	x
ApoN protein	Q2KIH2	x		x	x		x		
Beta-1,4-glucuronyltransferase 1	Q5EA01	x		x					
Selenoprotein P	P49907	x		x		x		x	x
SH3 domain-binding glutamic acid-rich-like protein 3 (Fragment)	G3X6S5	x							
Insulin-like growth factor II	P07456	x	x	x	x		x	x	x
Peptidyl-prolyl cis-trans isomerase A	P62935	x	x	x		x		x	
Uncharacterized protein	F1N514	x	x	x			x		
Adiponectin	Q3Y5Z3	x						x	
Fibromodulin	P13605	x		x		x			
Plasma serine protease	Q9N2I2	x	x	x	x	x	x	x	x

inhibitor									
SPARC related modular calcium binding 1	A0JNE0	x		x				x	
Beta-1,4-galactosyltransferase 1	P08037	x		x				x	
Chondroadherin	Q27972	x	x	x	x	x	x	x	x
Coactosin-like protein	Q2HJ57	x		x		x		x	
Elongation factor 1-alpha	E1B7J1	x		x		x		x	
Glutathione S-transferase Mu 1	E1BH17	x							
Histone H4	E1BBP7	x	x	x	x		x	x	x
Insulin-like growth factor-binding protein 2	F1N2P8	x	x			x	x	x	
Integrin-linked protein kinase	Q3SWY2	x		x		x		x	
Nucleoside diphosphate kinase B	Q3T0Q4	x							x
PDGFD protein	A4IFC0	x				x			
Protein AMBP	P00978	x	x	x	x	x	x	x	x
Ras suppressor protein 1	Q5E9C0	x		x		x		x	
Serotransferrin	Q29443	x	x	x	x	x		x	x
Ubiquitin-like modifier-activating enzyme 1	A3KMV5	x		x				x	
Uncharacterized protein	F1N6W9	x			x		x		
Uncharacterized protein (Fragment)	F1MVP0	x		x		x		x	
Keratin, type II cytoskeletal 5	M0QVZ6		x			x			
Kininogen-1	P01044	x	x	x	x	x	x	x	x
GLI pathogenesis-related 2	Q0VCH9		x				x		
Uncharacterized protein	E1BDY3		x	x			x	x	
Uncharacterized protein (Fragment)	E1B6Z6		x	x	x		x	x	
Apolipoprotein D	Q32KY0		x		x		x		x

Uncharacterized protein	F1N4M7		x	x			x		x
Proteasome subunit alpha type-7	Q3ZBG0		x	x			x	x	
Collagen alpha-1(I) chain	P02453		x				x		
Insulin-like growth factor-binding protein 3	P20959		x		x		x		x
Cytoplasmic tRNA 2-thiolation protein 2	Q3SZG9		x						
Collagen alpha-1(XII) chain	F1N401		x						
Brain ribonuclease	P39873		x				x		
Insulin-like growth factor-binding protein 4	Q05716		x					x	
Immunoglobulin J chain	Q3SYR8		x						
Pulmonary surfactant-associated protein B	P15781		x						
Tubulin alpha-4A chain	P81948			x	x			x	x
Kininogen-2	P01045			x			x	x	
4-trimethylaminobutyraldehyde dehydrogenase	F1N2L9			x		x		x	
Heat shock protein HSP 90-beta	Q76LV1			x	x		x	x	
Uncharacterized protein	G3N0B6			x		x		x	
CD14 protein	A6QNL0			x				x	
Proteasome subunit beta type-6	Q3MHN0			x				x	

Secreted frizzled-related protein 3	Q95117			x				x	
Glutathione S-transferase A1	Q28035			x					
Uncharacterized protein	F1N102			x	x			x	
Phosphoglycerate kinase 1	Q3T0P6			x				x	
Cytoplasmic aconitate hydratase	Q0VCU1			x					
L-lactate dehydrogenase A chain	P19858			x	x	x	x	x	x
Bifunctional purine biosynthesis protein PURH	Q0VCK0			x				x	
Protein-lysine 6-oxidase	P33072			x					
Proteasome subunit alpha type-4	Q3ZCK9			x					
Uncharacterized protein	G8JKW7			x	x			x	
Uncharacterized protein	Q08DQ6			x					
Cadherin-1	Q6R8F2				x		x		x
Ribosomal protein S4, Y-linked 1	A2VE06					x		x	
Complement C1q subcomponent subunit B	Q2KIV9					x			
Glutathione S-transferase	F1MX44					x			
Non-muscle caldesmon	F1MLW0						x		
KRT33A protein	A5PJJ1							x	
KRT82 protein	A3KMY1							x	

Tubulin beta-2B chain	E1BJB1							x	
Insulin-like growth factor-binding protein 6	F1MUK3							x	
Cysteine and glycine-rich protein 1	Q3MHY1							x	

Supplementary Table 3. Full hard corona profiles of silver NPs under various incubation conditions. Proteins identified in at least 2 of 3 replicas of silver nanoparticle samples incubated in cell culture media under various conditions. The samples labelling is, as follows: C for cubic, S for spherical silver nanoparticles, 1 for 1% FBS, 10 for 10% FBS incubation, 24 h for 24 hours incubation and 7 d for 7 days incubation. “x” marks the presence of the specific protein in the sample.

Experimental situation	Simulation model	% of Ag transformed into Ag_2S
Ag nanocubes, 7 days in RPMI with 1% FBS	60 nm particle, 4 nm Ag_2S layer	13.7 ± 0.7
	60 nm particle, 7 nm Ag_2S layer	15.3 ± 0.6
	60 nm particle, 10 nm Ag_2S layer	18.3 ± 0.9
Ag quasi-spheres 24 hours in RPMI with 1% FBS	70 nm particle, 4 nm Ag_2S layer	17.6 ± 0.5
	70 nm particle, 7 nm Ag_2S layer	19.6 ± 1.0
	70 nm particle, 10 nm Ag_2S layer	22.3 ± 1.5
Ag quasi-spheres 7 days in RPMI with 1% FBS	70 nm particle, 7 nm Ag_2S layer	37.8 ± 0.9
	70 nm particle, 10 nm Ag_2S layer	42.9 ± 1.4
Ag quasi-spheres 24 hours in RPMI with 10% FBS	70 nm particle, 4 nm Ag_2S layer	13.2 ± 0.5
	70 nm particle, 7 nm Ag_2S layer	14.6 ± 0.9
	70 nm particle, 10 nm Ag_2S layer	16.5 ± 1.4
Ag quasi-spheres 7 days in RPMI with 10% FBS	70 nm particle, 7 nm Ag_2S layer	28.8 ± 1.0
	70 nm particle, 10 nm Ag_2S layer	32.7 ± 1.5

Supplementary Table 4. Ag to Ag_2S quantified based on FDTD simulations. Amount of silver from Ag NPs transformed into Ag_2S upon incubation in cell culture media, estimated based on experimental plasmon shifts and calibration curves for peak shifts from FDTD simulated data.

Component	Concentration (mg L^{-1})	Component	Concentration (mg L^{-1})
Glycine	10.00	Biotin	0.20
L-Arginine	200.00	Cholin chloride	3.00
L-Asparagine	50.00	D-Calcium pantothenate	0.25
L-Aspartic acid	20.00	Folic acid	1.00
L-Cysteine	20.00	Niacinamide	1.00
L-Glutamic acid	20.00	Para-aminobenzoic acid	1.00
L-Histidine	15.00	Pyridoxine hydrochloride	1.00
L-Hydroxyproline	20.00	Riboflavin	0.20
L-Isoleucine	50.00	Thiamine hydrochloride	1.00
L-Leucine	50.00	Vitamin B12	0.0050
L-Lysine hydrochloride	40.00	i-Inositol	35.00
L-Methionine	15.00	$Ca(NO_3)_2 \cdot 4H_2O$	100.00
L-Phenylalanine	15.00	$MgSO_4 \cdot 7H_2O$	100.00
L-Proline	20.00	KCl	400.00
L-Serine	30.00	$NaHCO_3$	2000.00
L-Threonine	20.00	NaCl	6000.00
L-Tryptophan	5.00	Na_2HPO_4	800.00
L-Tyrosine	20.00	D-Glucose	2000.00
L-Valine	20.00	Gluthatione (reduced)	1.00

Supplementary Table 5. Cell culture media components. We use non-phenyl red RPMI-1640 (Invitrogen), supplemented with 1 % (by volume) Glutamax (Invitrogen) and 1 % (by volume) antibiotics (Penicillin-Streptomycin 5000 U ml^{-1}, Invitrogen). The composition of the un-supplemented cell culture medium, as provided by the supplier, is listed in the table.

**Compound**	**Molecular weight (g mole^{-1})**	**Formula**	**Reduced S atoms per molecule**	**Reduced S atoms per ml of RPMI-1640**
L-cysteine	240	$C_3H_7NO_2S$	1	8.0×10^{16}
L-methionine	149	$C_5H_{11}NO_2S$	1	6.1×10^{16}
Biotin	244	$C_{10}H_{16}N2O_3S$	1	4.9×10^{14}
Tiamine hydrochloride	337	$C_{12}H_{18}Cl_2N_4OS$	1	1.8×10^{15}
Gluthatione (reduced)	307	$C_{10}H_{17}N_3O_6S$	1	2.0×10^{15}

Supplementary Table 6. **Cell culture media components with reduced sulphur atoms.** List of RPMI-1640 compounds containing reduced sulphur atoms and their concentrations calculated based on the formula $C_S = N_S \cdot \frac{C \cdot 10^{-6}}{\mu} \cdot N_A$, where C_S is the number of reduced S atoms per ml of RPMI-1640, N_S is the number of reduced S atoms in the molecule of interest, C is the concentration of the molecule of interest, in mg L^{-1}, of RPMI-1640, μ is the molecular weight of the molecule of interest, expressed in g $mole^{-1}$ and N_A is Avogadro's constant. The main sources of reduced sulphur are L-cysteine and L-methionine.

	p value Ag NPs	p value partially-sulphidated Ag NPs	p value completely-sulphidated Ag NPs	p value Ag^+ ions
2 μg ml^{-1} Ag	$5.63*10^{-2}$	$7.24*10^{-1}$	$3.05*10^{-2}$	0 ***
5 μg ml^{-1}Ag	$3.06*10^{-1}$	$1.07*10^{-1}$	$2.75*10^{-1}$	0 ***
10 μg ml^{-1}Ag	$1.09*10^{-5}$ ***	$1.58*10^{-3}$ **	$2.77*10^{-1}$	0 ***
15 μg ml^{-1}Ag	$5.00*10^{-8}$ ***	$2.94*10^{-3}$ **	$6.99*10^{-1}$	0 ***
25 μg ml^{-1}Ag	$1.00*10^{-8}$ ***	$1.58*10^{-3}$ **	$8.11*10^{-1}$	0 ***
50 μg ml^{-1}Ag	0 ***	$4.25*10^{-1}$	$8.22*10^{-1}$	0 ***
100 μg ml^{-1}Ag	0 ***	$2.02*10^{-1}$	$8.00*10^{-1}$	0 ***

Supplementary Table 7. Statistical significance of MTT data. Two-tailed Students t-test p-values for data obtained from MTT experiments. n=6, statistically significant results are marked with **($p<0.005$) or ***($p<0.0005$). All sets of data exhibited normal distribution, with similar variance between groups.

Supplementary Discussion

Formation and separation of hard and soft protein coronas

When nanoparticles come in contact with a biological environment, they interact with biomolecules which form what are now known as coronas around the particles. The main constituents of these coronas are proteins, which is why they are often referred to as "protein coronas"; they are classified as "hard" (or long-lived, slowly-exchanging) and "soft" (or short-lived, rapidly-exchanging) depending on various parameters which will be briefly discussed here.

In a medium such as serum thousands of different types of proteins are present, in concentrations spanning over several orders of magnitude[2]. Upon introduction of a nanoparticle in such an environment, the most abundant proteins will rapidly reach the particle surface and bind to it. However, they may not be the biomolecules with the highest affinity for that surface, therefore over time they will be replaced by proteins with higher affinity, but lower mobility, which take longer to reach the nanoparticle. This behaviour is known as the Vroman effect[3,4]. The affinity of a protein for a certain nanoparticle surface depends on parameters such as particle type[5,6], surface chemistry[7,8], particle size[7,9-11] and shape[6].

Furthermore, corona formation is an equilibrium process, which also depends on the initial concentration of biomolecules in the system, as well as the mean residence time of each protein at the particle surface and inter-protein interactions such as, for instance, cooperative binding. These parameters have been discussed in detail elsewhere[12,13], and they are important in distinguishing between hard and soft coronas[14].

In practice, in experimental settings such as the ones described in this paper and in several protein corona studies[7,10,15], nanoparticles are incubated in plasma/serum-containing media for a certain amount of time. Following incubation, several rounds of washing take place through centrifugation, removal of supernatants and resuspension of particles in water or a protein-free buffer. The first washing step removes the unbound proteins and all the loosely-bound proteins whose mean residence time at the particle surface is shorter than the centrifugation step (15 minutes in our case). Resuspension of the pelleted particles in water or buffer changes the biomolecule equilibrium which, for some proteins, means they will detach from the nanoparticles and move into the bulk. Centrifugation results, again, in removal of the proteins that have become free, as well as the loosely-bound biomolecules with residence times shorter than the centrifugation step. The hard corona is comprised of all the proteins that

are still bound to the particle surface after repeated washing, while the soft corona proteins are those which were bound (to the particle surface or the hard corona[15]) at the end of the incubation, but were removed during several rounds of centrifugation and resuspension. The centrifugation time and speed, as well as the number of repeats of this washing process depend on the particle type and of the protein concentration in the incubation medium. The proteins that cannot be removed through repeated washing by centrifugation and resuspension in water form what is known as the hard corona. These proteins can only be detached from the surface of nanoparticles using harsh treatments such as boiling in a mixture of surfactant and reducing agents or denaturation in concentrated urea solutions or enzymatic digestion.

The long-lived nature and strong-binding of the hard corona have been proven by experts in the field in studies showing this hard corona provides a protein fingerprint that can be used to trace particles passing from one environment to another, as the hard corona conserves many of the components acquired in the initial incubation environment[16,17]. Furthermore, it has been shown that the hard protein corona is retained during intracellular trafficking[18] and is only degraded when the particles are exposed to the low pH harsh conditions inside lysosomes[19].

Cytokine production and quantification

As discussed in the main paper, both pristine and partially-sulphidated Ag NPs increase TNFα and MIP-2 production 3-fold. Here we see that IL-1β is only slightly increased by a high concentration (50 μg ml^{-1}) of Ag NPs after partial-sulphidation. IL-6 production is increased at 50 μg ml^{-1} particle dose by both partially and completely-transformed NPs, in agreement with the observations for TNFα which, together with IL-6, is an early marker of inflammation[20]. It should be noted that for the Ag NPs 50 μg ml^{-1} is a lethal dose, with about 12 % cell survival (Figure 5), so cytokine release profiles may not be comparable to those at sub-lethal values. IL-18 concentrations are only measurable for the sulphidated NPs treatments and an increase as compared to controls is observed only for the highest concentration (100 μg ml^{-1}) of partially transformed Ag NPs. Calibration curves for all measured cytokines are provided in Supplementary Fig. 19.

Supplementary Methods

Incubation in cell culture media

Silver nanocubes were incubated (37 °C, 5 % CO_2) in RPMI-1640 medium, without any added serum, for 1 hour and 24 hours. Transmission electron microscopy (TEM) images can be seen in Supplementary Fig 1, showing that at 24 hours silver sulphide is present. At 1 hour, however, there is no nano-Ag_2S at the surface of the metal nanoparticles. We have previously shown that, upon incubation in serum-containing RPMI-1640 cell culture medium, the polyvinylpyrrolidone (PVP) coating around the nanocubes is already replaced by proteins after 1 hour[1]. As such, the sulphidation of silver nanoparticles in foetal bovine serum (FBS) containing cell culture medium occurs with proteins and not polymer present at the surface of the Ag nanoparticles (NPs).

SDS-PAGE of serum hard coronas

Silver nanocubes with diameters ranging from 50 to 88 nm (as determined by SPIP™ analysis of TEM images) were incubated (24 hours, 37 °C, 5 % CO_2) in RPMI-1640 cell culture medium supplemented with either 1 % or 10 % FBS. Following incubation, unbound and loosely-bound serum proteins were removed through several rounds of centrifugation using a Heraeus Multifuge X1R table top centrifuge (Thermo Scientific) and re-suspension in MilliQ water. Strongly-bound, hard corona biomolecules were detached from the particles by boiling in Pierce® lane marker reducing sample buffer (Thermo Scientific) and the resulting charged proteins were separated by SDS-PAGE on a 10-well Pierce® 4-20 % polyacrylamide precast gel (Thermo Scientific). The proteins were visualised by staining with Imperial Protein Stain (Thermo Scientific) and the gel was scanned on a Gel DocTM EZ Imager (Bio-Rad) using a White Light Sample Tray (Bio-Rad). The PageRuler unstained protein ladder (Thermo Scientific) was used as a molecular weight standard (first lane in the gel in Supplementary Fig. 5). While the bands for the samples incubated in 10 % FBS are somewhat more intense, the same bands are present in all samples, regardless of the particle size and serum concentration

Mass Spectrometry of long-lived protein coronas of Ag NPs

Cubic and quasi-spherical PVP-coated Ag NPs were incubated for 24 hours or 7 days in RPMI-1640 cell culture medium supplemented with either 1 % or 10 % FBS. Subsequently,

the particles were washed to remove unbound and loosely-bound proteins. The washing procedure involved repeated centrifugation and re-suspension steps, as described above.

Hard corona proteins were extracted from the nanoparticles by sequential washing with 0.5 % formic acid and 6 M urea. The combined extract was lyophilized, dissolved in 200 mM ammonium bicarbonate and reduced by the addition of 10 mM dithiothreitol. After 30 minutes, the samples were alkylated by addition of 30 mM iodoacetamide. Finally, the samples were diluted 3 times with 200 mM ammonium bicarbonate to lower the urea concentration to 2 M and treated with trypsin for 16 hours at 37 °C. The resulting peptides were micro purified and analysed by liquid chromatography-tandem mass spectrometry (LC-MS/MS). Triplicates were prepared for each particle type and incubation condition.

nLCI-MS/MS analysis was performed on an EASY-nLC II system (ThermoScientific) connected to a TripleTOF 5600 mass spectrometer (AB Sciex) equipped with a NanoSpray III source (AB Sciex) operated under Analyst TF 1.5.1 control. The micropurified sample was suspended in 0.1 % formic acid, injected, trapped and desalted on a 2 cm x 100 μm Trap column packed in-house with RP ReproSil-Pur C18-AQ 3 μm resin (Dr. Marisch GmbH, Ammerbuch-Entringen, Germany). The peptides were eluted from the trap column and separated on a 15-cm analytical column (75 μm i.d.) packed in-house in a pulled emitter with RP ReproSil-Pur C18-AQ 3 μm resin (Dr. Marisch GmbH, Ammerbuch-Entringen, Germany). Peptides were separated using a 50 min gradient from 5 % to 35 % phase B (0.1 % formic acid and 90 % acetonitrile) and a flow rate of 250 nl min^{-1}.

All raw MS files were processed using Mascot Distiller (Matrix Science) using the default settings from the ABSciex_5600.opt file except that the MS/MS Peak Picking “Same as MS Peak Picking” was deselected and “Fit method” was set to “Single Peak”. After peak picking all scans, a search against the Uni-prot database (Proteome UP000009136) using the Mascot search engine (matrix science) was performed. Search parameters were set with carbamidomethyl as fixed modification and methionine oxidation as variable modification and allowing one miscleavage. Peptide tolerance and MS/MS tolerance were set to 10 ppm and 0.1 Da respectively. The relative amounts of the identified proteins were calculated using an average [MD] quantitation protocol. Quantification settings had a significance threshold at 0.01, number of peptides used for quantitation was 3, matched rho was 0.8, XIC threshold was 0.3 and isolated precursor threshold was set at 0.7. Protein intensities were normalized to the total protein intensity of the analysis. The average relative protein amount and standard deviation was calculated based on the normalized intensities in three replicates. The major proteins quantified in the Ag NP hard coronas are listed in Supplementary Table 1 (cubes) and

Supplementary Table 2 (spheres). Values are expresses as % of total intensity. The full list of hard corona proteins bound to various investigated Ag NPs is available in Supplementary Table 3.

Nanoparticle Tracking Analysis for particle size measurement

Cubic and quasi-spherical Ag NPs were incubated (24 hours and 7 days) in RPMI-1640 cell culture medium supplemented with 1 % or 10 % FBS. The silver concentration during incubation was the same as for the TEM and MS studies. After incubation, unbound proteins were removed by centrifugation and the NPs were re-suspended in MilliQ water and then diluted such as to ensure a concentration of $\approx 10^9$ particles ml^{-1}. Measurements of Ag NPs hydrodynamic diameter were performed using a Nanoparticle tracking analysis equipment (NTA, NanoSight LM10-HS, NanoSight Ltd., UK) with the NanoSight software, version 3.0. The results are presented in Supplementary Fig. 6. Recordings of 60 seconds each were acquired in triplicate for every sample.

UV-Vis spectra of silver nanocubes in BSA

UV-vis spectra of silver nanocubes incubated in 0.4 mg ml^{-1} or 4 mg ml^{-1} BSA were collected in the range of 300 to 800 nm using a Shimadzu UV-visible-NIR UV-3600 spectrophotometer. Measurements were performed in triplicate for each of the three samples prepared for the two protein concentrations, before and after washing of unbound and loosely-bound proteins by centrifugation of particles followed by re-suspension in phosphate buffered saline. As previously published, blue shifting of the peak position upon washing would indicate soft corona removal[21], if that corona was initially present. Here, we see no such shifts (Supplementary Fig. 8), proving the absence (as expected) of BSA soft coronas.

Ion release of silver nanocubes in cell culture media

Silver nanocubes (2 and 10 μg ml^{-1}) were incubated in RPMI-1640 supplemented with 1 % or 10 % FBS for 1 or 7 days. After incubation, undissolved particles were separated by spinning down the suspension (30 minutes, 16000 g). The released silver ions, which remained in the liquid phase, were analysed by flame atomic absorption spectroscopy (F-AAS) on a PerkinElmer Analyst 300 atomic absorption spectrometer mounted with a silver lumina hollow cathode lamp (PerkinElmer, Denmark), after dilution of the supernatant with 5 % HNO_3. Triplicate samples were prepared for each incubation condition and duplicate F-AAS samples were measured for each incubation sample.

Finite-difference time-domain (FDTD) simulations and interpretation

Finite-difference time-domain simulations were performed using the FDTD Solutions software (Lumerical Solutions, Inc.). The dielectric properties of silver were represented by a numerical fit to experimental data[22] while constant refractive index (RI) values were used to model the environment. The constant RI values were calculated, considering a layer of specific thickness (4, 7 or 10 nm) around the particle and various degrees of occupancy (x) by silver sulphide, using the formula RI = 2.2*x + (1-x)*1.333, with 2.2 being the refractive index of Ag_2S and 1.333 that of pure water. A single plasmonic particle was studied using non-periodic boundary conditions in combination with Perfectly Matched Layers. Uniform meshing with size of 1×1×1 nm was used in the region containing the nanoparticle.

For a given set of conditions (particle size, layer thickness), FDTD simulations of plasmon shifts of Ag NPs depending on increasing RIs due to accumulation of Ag_2S result in a calibration curve. Comparing experimentally measured shifts with that calibration curve, we assess the percentage of sulphide present in a layer of known thickness and, hence, known volume. Considering the density of Ag_2S we calculate the amount of sulphide around one Ag NP for the given conditions, which we then multiply by the total number of silver nanoparticles, thus obtaining the total amount of sulphide. We express this as percentage of transformed silver, knowing the mass we introduced in the system at the beginning of the incubation.

FDTD simulations were employed to obtain spectra of 60 and 70 nm spherical particles, surrounded by a 4, 7, or 10 nm thick layer of Ag_2S with various degrees of sulphide occupancy (0-100 %), as can be seen in Supplementary Fig. 16 A-C, G-I. Peak shifts at the maximum dipole absorbance were used to obtain calibration curves for each setting (particle size and layer thickness). The calibration curves were used to assess the amount of silver transformed into silver sulphide for various incubation times and serum contents, considering that all Ag NPs are of the same size. The results are presented in Supplementary Table 4.

Layer thicknesses were chosen based on the protein hard-corona model previously described[1] and on TEM observations of Ag_2S at our nanoparticles. The shifts in the position of the maximum absorbance peak were employed for calibration curves (Supplementary Fig. 16 D-F, J-L), which were subsequently used to estimate the amount of silver transformed into sulphide based on the experimentally observed peak shifts. Similar amounts of Ag_2S were obtained for a given particle size regardless of the chosen layer thickness, with the numerical values for all the studied settings being presented in Supplementary Table 4. Overall, about 15-20 % of the silver in the quasi-spherical particles is transformed into sulphide at 24 hours

incubation in 1 % or 10 % FBS, with the values increasing to 30-40 % at 7 days. In this case, the theoretical model employed for simulations was that of a 70 nm spherical nanoparticle, with the diameter chosen based on the experimental values for the metal core obtained from TEM images.

Supplementary Fig. 17 shows the simulated data and calibration curve for a 60 nm Ag NP with a 7 nm layer of Ag_2S, at various degrees of layer occupancy by the sulphide, ranging from 0 % to 100 %. For a cube and a sphere of 60 nm diameter, the simulations in Supplementary Fig. 17C show a ≈ 10 nm blue peak shift upon rounding of the edges and corners. This suggests that our experimental shifts for the cubes are underestimated, as these particles gradually become more spherical upon incubation in serum-containing media. The shape changes that occur throughout the incubation do not allow for precise FDTD models, so the size and shape of the particle at the end of the incubation (7 days in 1 % FBS) based on TEM images were considered as a starting point for the simulations in Supplementary Fig 16A. We simulated a spherical nanoparticle with a diameter of 60 nm, similar to that of the cube turned into a sphere after 7 days in 1 % serum, and with increasing amounts of sulphide in the surrounding layer. The thickness of the layer (4, 7 or 10 nm) did not impact the final estimations of sulphide content, as can be seen in Supplementary Table 4. The experimentally obtained plasmon shifts from UV-vis spectra were increased by 10 nm to account for the underestimation introduced by the particle reshaping, and, using the calibration curves in Supplementary Fig. 17B and 16 (D-F) to calculate how much space around the particles is occupied by Ag_2S, we estimated that about 15 % of the silver in the nanocubes is transformed into sulphide after 7 days incubation in 1 % FBS.

MTT assay

The cellular mitochondrial activity was measured using MTT assays, with minor modifications to a previously described method[23]. Briefly, the cells were seeded at 2 x 10^4 cells per well. They were exposed to silver ions, PVP-coated Ag nanocubes, partially and completely-sulphidated silver nanocubes, all at concentrations of 0, 2, 5, 10, 15, 25, 50 and 100 μg ml^{-1} in RPMI-1640 supplemented with 10 % FBS and incubated for 24 hours at 37 °C and 5 % CO_2. Following incubation, the test medium was collected and used for cytokine analysis. The cells were incubated with 100 μl of MTT solution (0.5 mg ml^{-1} MTT diluted in phenol red free RPMI-1640 medium without FBS) for 2 hours at 37 °C and 5 % CO_2. Subsequently, the MTT solution was discarded and DMSO (100 μl) was added to the every

well. A microplate reader (EL800, Bio-Tek Instruments, Inc.) was used to read the optical density (OD) at 550 nm, with a reference at 655 nm. The cell viability for each treatment was calculated as the ratio of the mean OD of separate wells (n=6) relative to that of the control, where only cell culture medium was added.

Multiplex assay for cytokine quantification

J774 cells were seeded in 96-well plates and exposed to Ag^+ ions, Ag NPs, partially and completely sulphidated Ag NPs, as described above. After 24 hours incubation, the supernatants were transferred into Eppendorf tubes and centrifuged for 10 minutes at 20,000 g in order to pellet the Ag NPs. Mouse cytokines were analysed using the magnetic bead-based ProcartaPlex Mouse Th1/Th2 cytokine panel (GM-CSF, IFNγ, IL-1β, IL-2, IL-4, IL-5, IL-6, IL-12p70, IL-13, IL-18, TNFα) which was supplemented with ProcartaPlex Mouse IL-1α, IL-10 and MIP-2 Simplex, all supplied by eBioscience. The assay was performed following the manufacturer's specifications and the results were quantified using the Bio-Plex® MAGPIX™ Multiplex Reader.

Supplementary References

1. Wang, Y., Zheng, Y., Hunag, C.Z. & Xia, Y. Synthesis of Ag nanocubes 18-32 nm edge length: the effects of polyol on reduction kinetics, size control, and reproducibility. *J. Am. Chem. Soc.* **135**, 1941-1951 (2013).
2. Anderson, N.L & Anderson, N.G. The human plasma proteome. History, character and diagnostic prospects. *Mol. Cell. Proteomics* **1**, 845-867 (2001).
3. Casals, E. & Puntes V.E. Inorganic nanoparticle biomolecular corona: formation, evolution and biological impact. *Nanomedicine-UK* **7**, 1917-1930 (2012).
4. Vroman, L. Effect of adsorbed proteins on the wettability of hydrophilic and hydrophobic solids. *Nature* **196**, 476-477 (1962).
5. Monopoli, M.P. et al. Physical-chemical aspects of protein corona: relevance to in vitro and in vivo biological impacts of nanoparticles. *J. Am. Chem. Soc.* **133**, 2525-2534 (2011).
6. Deng, Z.J. et al. Differential plasma protein binding to metal oxide nanoparticles. *Nanotechnology* **20**, 455101-455109 (2009).
7. Lundqvist, M. et al. Nanoparticle size and surface properties determine the protein corona with possible implications for biological impacts. *P. Natl. Acad. Sci. USA* **105**, 14265-14270 (2008).
8. Jedlovsky-Hajdú, A., Baldelli Bombelli, F., Monopoli, M.P., Tombácz, E. & Dawson, K.A. Surface coatings shape the protein corona of SPIONs with relevance to their application in vivo. *Langmuir* **28**, 14983-14991 (2012).
9. Dobrovolskaia, M.A. et al. Interactions of colloidal gold nanoparticles with human blood: effects on particle size and analysis of plasma protein binding profiles. *Nanomed, Nanotech. Biol. Med.* **5**, 106-117 (2009).
10. Tenzer, S. et al. Nanoparticle size is a critical physic-chemical determinant of the human blood plasma corona: a comprehensive quantitative proteomic analysis. *ACS Nano* **5**, 7155-7167 (2011).
11. Schäffler, M. et al. Serum protein identification and quantification of the corona of 5, 15 and 80 nm gold nanoparticles. *Nanotechnology* **24**, 265103-265111 (2013).
12. Del Pino, P. et al. Protein corona formation around nanoparticles – from the past to the future. *Mater. Horiz.* **1**, 301-313 (2014).

13. Walkey, C.D. & Chan, W.C.W. Understanding and controlling the interaction of nanomaterials with proteins in physiological environment. *Chem. Soc. Rev.* **41**, 2780-2799 (2012).
14. Cedervall, T. et al. Understanding the nanoparticle-protein corona using methods to quantify exchange rates and affinities of proteins for nanoparticles. *P. Natl. Acad. Sci. US* **104**, 2050-2055 (2007).
15. Casals, E., Pfaller, T., Duschl, A., Oostingh, G.J. & Puntes, V. Time evolution of the nanoparticle protein corona. *ACS Nano* **4**, 3623-3632 (2010).
16. Lundqvist, M. et al. The evolution of the protein corona around nanoparticles: A test study. *ACS Nano* **9**, 7503-7509 (2011).
17. Monopoli, M.P., Åberg, C., Salvati, A. & Dawson, K.A. Biomolecular coronas provide the biological identity of nanosized materials. *Nat. Nanotechnol.* **7**, 779-786 (2012).
18. Bertoli, F. et al. Magnetic nanoparticles to recover cellular organelles and study time resolved nanoparticle-cell interactome through uptake. *Small* **10,** 3307-3315 (2014).
19. Wang, F. et al. The biomolecular corona is retained during nanoparticle uptake and protects cells from the damage induced by cationic nanoparticles until degraded in the lysosomes. *Nanomed.-Nanotechnol.* **9**, 1159-1168 (2013)
20. Bopst, M., Haas, C., Car, B. & Engster H.P. The combined inactivation of tumor necrosis factor and interleukin-6 prevents induction of the major acute phase proteins by endotoxin. *Eur. J. Immunol.* **28**, 4130-4137 (1998).
21. Miclăuş, T., Bochenkov, V.E., Ogaki, R., Howard, K.A. & Sutherland D.S. Spatial mapping and quantification of soft and hard protein coronas at silver nanocubes. *Nano Lett.* **14**, 2086-2093 (2014).
22. Hagemann, H.-J., Gudat, W. & Kunz, C. Optical constants from the far infrared to the x-ray region: Mg, Al, Cu, Ag, Au, Bi, C, and Al_2O_3. *J. Opt. Soc. Am.* **65**, 742-744 (1975).
23. Mosmann, T., Rapid colorimetric assay for cellular growth and survival: application to proliferation and cytotoxicity assays. *J. Immunol. Methods.* **65**, 55-63 (1983).
